# The MAP Kinase p38 Is Part of *Drosophila melanogaster's* Circadian Clock

**DOI:** 10.1371/journal.pgen.1004565

**Published:** 2014-08-21

**Authors:** Verena Dusik, Pingkalai R. Senthilan, Benjamin Mentzel, Heiko Hartlieb, Corinna Wülbeck, Taishi Yoshii, Thomas Raabe, Charlotte Helfrich-Förster

**Affiliations:** 1 Neurobiology and Genetics, Biocenter, University of Würzburg, Würzburg, Germany; 2 Institute of Medical Radiation and Cell Research, University of Würzburg, Würzburg, Germany; 3 Institute of Zoology, University of Regensburg, Regensburg, Germany; 4 Graduate School of Natural Science and Technology, Okayama University, Okayama, Japan; Charité - Universitätsmedizin Berlin, Germany

## Abstract

All organisms have to adapt to acute as well as to regularly occurring changes in the environment. To deal with these major challenges organisms evolved two fundamental mechanisms: the p38 mitogen-activated protein kinase (MAPK) pathway, a major stress pathway for signaling stressful events, and circadian clocks to prepare for the daily environmental changes. Both systems respond sensitively to light. Recent studies in vertebrates and fungi indicate that p38 is involved in light-signaling to the circadian clock providing an interesting link between stress-induced and regularly rhythmic adaptations of animals to the environment, but the molecular and cellular mechanisms remained largely unknown. Here, we demonstrate by immunocytochemical means that p38 is expressed in *Drosophila melanogaster's* clock neurons and that it is activated in a clock-dependent manner. Surprisingly, we found that p38 is most active under darkness and, besides its circadian activation, additionally gets inactivated by light. Moreover, locomotor activity recordings revealed that p38 is essential for a wild-type timing of evening activity and for maintaining ∼24 h behavioral rhythms under constant darkness: flies with reduced p38 activity in clock neurons, delayed evening activity and lengthened the period of their free-running rhythms. Furthermore, nuclear translocation of the clock protein Period was significantly delayed on the expression of a dominant-negative form of p38b in *Drosophila's* most important clock neurons. Western Blots revealed that p38 affects the phosphorylation degree of Period, what is likely the reason for its effects on nuclear entry of Period. *In vitro* kinase assays confirmed our Western Blot results and point to p38 as a potential “clock kinase” phosphorylating Period. Taken together, our findings indicate that the p38 MAP Kinase is an integral component of the core circadian clock of *Drosophila* in addition to playing a role in stress-input pathways.

## Author Summary

The circadian and the stress system are two distinct physiological systems that help the organism to adapt to environmental challenges. While the latter elicits reactive responses to acute environmental changes, the circadian system predicts daily occurring alterations and prepares the organism in advance. However, these two responses are not mutually exclusive. Studies in the last years prove a strong interaction between both systems showing a strong time-related stress response depending on the time of day of stressor presentation on the one hand and increased disturbances of daily rhythms, like sleep disorders, in consequence of uncontrolled or excessive stress on the other. Here, we show that the mitogen-activated protein kinase p38, a well characterized component of immune and stress signaling pathways is simultaneously a part of the core circadian clock in *Drosophila melanogaster*. Our results demonstrate that p38 is activated in a circadian manner and that under constant darkness normal p38 signaling is necessary for the maintenance of 24 h rhythms on the behavioral and molecular level. Together, this strongly indicates a role of p38 in the core clock and further suggests that it is a possible nodal point between the circadian and the stress system.

## Introduction

Circadian clocks provide a key advantage to organism allowing them to prepare in advance for daily environmental changes. They control daily rhythms in physiology and behavior, as locomotor activity, sleep-wake cycles and hormonal secretion. A hallmark feature of these clocks is that they oscillate with free-running periods of ∼24 h, even in absence of external time cues. Molecularly, circadian clocks depend on species-specific clock genes and proteins that interact in complex feedback loops to rhythmically control gene transcription (reviewed in [1]–[2]). However, research of the last few years demonstrated that not only rhythmic gene expression but also post-translational modifications, especially protein phosphorylation, play a crucial role in generating and maintaining circadian rhythms and most importantly in determining the speed of the oscillations [3]–[9].

Studies in *Drosophila melanogaster* have been instrumental in our understanding of clock mechanisms in general and mammalian ones in particular. In *Drosophila's* main feedback loop, the core clock genes *period* (*per*) and *timeless* (*tim*) are rhythmically transcribed and translated into the proteins PER and TIM. Following phosphorylation by kinases (and/or dephosphorylation by phosphatases), both proteins accumulate in the cytoplasm and finally translocate back to the nucleus to inhibit their own transcription as well as that of clock-controlled genes (reviewed in [10]). Even if most of the clock proteins are phosphorylated within this molecular machinery, PER seems to be the clock component behaving as the primary “phospho-timer” [4], [6]. Recent findings indicate that PER proteins in animals possess up to 25–30 phosphorylation sites [5], [11] many of which undergo daily changes in phosphorylation. These temporal changes in PER phosphorylation are crucial for a functioning clock, since they modulate the stability of PER as well as the time of its nuclear entry, and in this way determine the pace of the clock [11]–[13]. While in the past it was thought that the amount of phosphate residues of clock proteins determines their degradation, studies nowadays show that it is rather site-directed phosphorylation that modulates clock protein function and stability [11]–[14]. So far, in *Drosophila* just a few kinases have been identified that interact with PER: DBT [15]–[17], SGG [12], CK2 [18]–[20] and proline-directed kinases as NEMO/NLK [12]–[13]. The latter belong to the CMGC family of kinases that also includes the evolutionarily conserved superfamily of mitogen-activated protein kinases (MAPKs) [21].

Sanada et al. [22] consider that mammalian extracellular signal-regulated kinase (ERK), a member of the MAPK superfamily, function in the circadian system either regulating biochemical activities and stabilities of clock components via phosphorylation or mediating coupling of pacemakers among clock cells. Interestingly, the modulation (phosphorylation) mechanism in *Drosophila's* core clock was only recently linked to MAPK signaling pathways. Several studies in *Drosophila* reported an ERK-binding domain in the kinase S6KII, a homologue of the mammalian p90 ribosomal S6 kinase (RSK), and claimed the importance of this ERK-binding domain for the interaction of S6KII with CK2 and the modulation of circadian behavior [23]–[24]. These findings strongly point to an involvement of MAPKs in the circadian clock of organisms.

The MAP Kinase p38 is a serine/threonine kinase that is activated by a variety of external stressors, including changes in osmolarity, heat shock and UV-irradiation [25]–[26]. Like all MAPKs, p38 contains a canonical TGY dual phosphorylation motif and requires phosphorylation of both the Thr184 and Tyr186 residue to achieve full enzymatic activity [25]. Intensive research in the last years revealed a wide spectrum of both nuclear and cytoplasmatic targets of p38, ranging from transcription factors like Mef2 [27] and ATF2 [28]–[29], growth factors and regulatory cell cycle proteins [30]–[31] to a limited number of subordinate kinases, such as MK2 [32]–[33], CK2 [34]–[35] and MSK [36]. Considering the variety and diversity of p38 targets, an extent and complex signaling network arises that regulates diverse cellular processes depending on cell type, tissue and stimuli.

The complexity of this p38 MAPK signaling network becomes even more elaborate as many cells express diverse isoforms of p38. The genome of the fruit fly encodes two functional p38 orthologues - p38a and p38b [25]–[26]. Phosphorylation of both is well described in respect of *Drosophila* development [27]–[28], [37], stress and immune response [25]–[27], [38]–[40]. Interestingly, various studies in mammals [41]–[42] and fungi [43]–[44] additionally revealed a light-dependent as well as circadian activation of p38 and further linked this to a role within the circadian system. This link is very interesting, since at least in mammals the stress system and circadian system are mutually linked [45]–[46]. Furthermore, as stated above phosphorylation of the core clock proteins is a crucial step in circadian rhythm generation in all organisms, and MAPKs could potentially participate in this process. Nevertheless, the function of p38 MAPK within the circadian clock remains largely unknown.

Here, we show for the first time p38 MAPK expression in *Drosophila* clock neurons and further confirm a darkness- and clock-dependent activation of p38 in these cells. Behavioral data of flies with modified p38 levels in clock neurons clearly indicate a role for p38 MAPK signaling in wild-type timing of evening activity in LD 12∶12 (12 hours light: 12 hours darkness) as well as in maintaining 24 h behavioral rhythms in constant conditions. The observed behavioral effects are consistent with a delayed nuclear entry of PER in flies expressing a dominant negative form of p38b, even placing p38 function into the core circadian clock. Finally, Western Blot analysis and *in vitro* kinase assays give first hints that p38 might modulate circadian rhythmicity by phosphorylating PER.

## Results/Discussion

### p38 MAPK localizes in clock neurons

**Figure 1:**
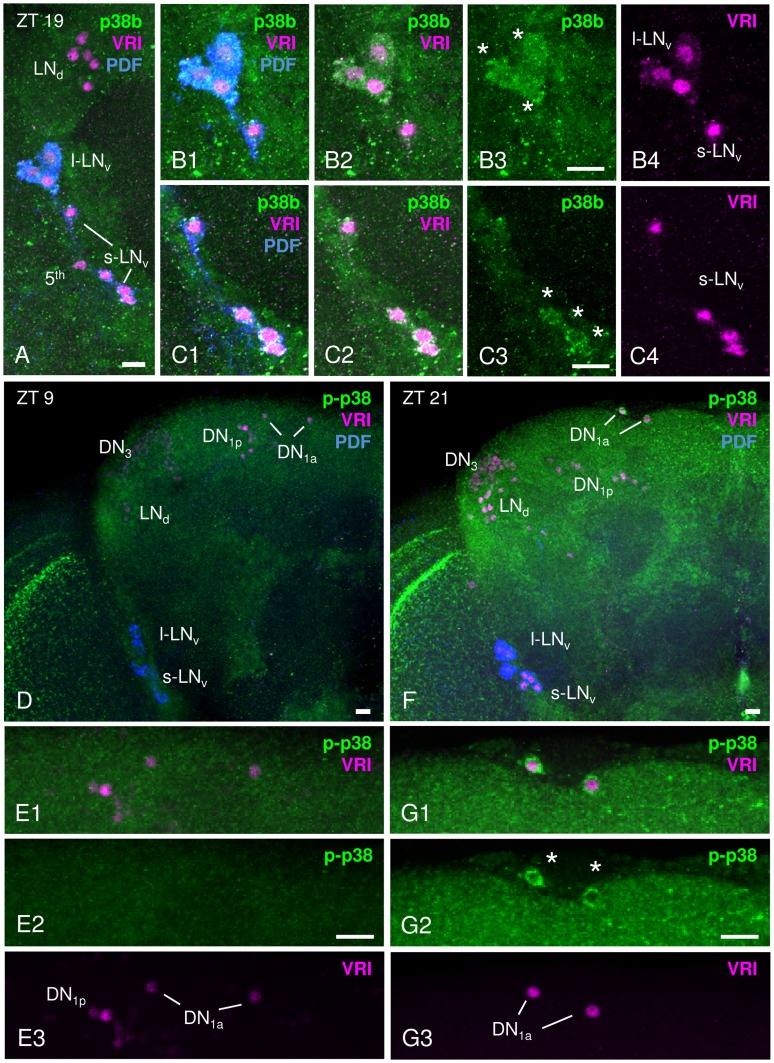
p38 MAPK expression pattern in adult male *Canton S* brains. p38 MAPK distribution within the circadian clock was investigated immunohistochemically with an antibody directed against *Drosophila* p38b (A–C) and against phosphorylated human p38 (D–G). A–C: Staining with anti-p38b (green) in *Canton S* wildtype brains was visible in many cell bodies close to the lateral clock neurons, but co-labeling with anti-VRI (magenta) and anti-PDF (blue) revealed clear p38b expression in the l-LN_v_s (white stars in B3) and the s-LN_v_s (white stars in C3). B1–B4 represents a close-up of l-LN_v_s, C1–C4 a shows close-up of s-LN_v_s. Furthermore, we found staining in the entire cortex including the region of the dorsal neurons (see Fig. S1). D–G: Staining with anti-p-p38 (green) was restricted to fewer neurons, but revealed again staining in the entire cortex that was stronger at night (F, ZT21) than during the day (D, ZT9). Double-labeling with anti-VRI (magenta) and anti-p-p38 antibody (green) revealed active p38 only in 2 clock neurons, the DN_1a_s (white stars in G2). Also in these cells, p-p38 staining intensity depended on the time of day, showing a higher level of active p38 at ZT 21 (G2, white stars) than at ZT9 (E2). E1–E3 and G1–G3 represent a close-up of DN_1a_s. Scale bar = 10 µm.

Although p38 MAPK is expressed in the hamster SCN [41] and regulates the chick pineal circadian clock [42], expression in the fly's clock has not been reported so far. The endogenous clock of *Drosophila* consists of approximately 150 clock neurons in the brain that are largely subdivided into 9 subgroups: small ventral lateral neurons (s-LN_v_s), large ventral lateral neurons (l-LN_v_s), 5^th^ small ventral lateral neuron (5^th^ s-LN_v_), dorsal lateral neurons (LN_d_s), 4 clusters of dorsal neurons (DN_1a_s, DN_1p_s, DN_2_s and DN_3_s) and lateral posterior neurons (LPNs) [47]–[49]. To investigate whether the clock neurons utilize p38 MAPK signaling pathways, we did immunohistochemistry on adult brains using the enhancer trap line *p38b-Gal4* in combination with a *UAS-GFP* transgene. GFP-expressing brains were immunolabelled with anti-GFP, anti-PER and anti-PDF at ZT21 (3 h before lights-on), when PER is mainly nuclear. Interestingly, *p38b*-driven GFP showed a broad expression within the brain as reported in Vrailas-Mortimer et al. [27] and colocalized with anti-PER and anti-PDF in at least four clock neurons, the large ventral lateral neurons (l-LN_v_, Fig. S1). Although, we were not able to reliably co-stain more clock neurons, our p38b-Gal4-staining pattern suggests that p38 is likely expressed in further clock neurons. To verify this we performed p38 antibody staining on *Canton S* wildtype brains using three different antibodies – two raised against *Drosophila* p38 (not distinguishing between the isoforms and between active/phosphorylated and inactive/unphosphorylated p38) and one raised against the dually phosphorylated isoforms of human p38 recognizing also phosphorylated *Drosophila* p38 (Cell Signaling Technology). The two *Drosophila* p38 antibodies, p38b (kindly provided by T. Adachi-Yamada) and p38 (Santa Cruz Biotechnologies), gave rather broad staining with several cell bodies labeled in the region of the clock neurons resembling the staining pattern of the *p38b*-driven GFP (Fig. 1A–C; Fig. S1). Double-labeling with anti-VRI and anti-PDF showed that both antibodies reliably labeled the PDF-positive l-LN_v_s as well as the PDF-positive s-LN_v_s (as depicted for anti-p38b in Fig. 1A–C). In addition, there was staining in the entire cortex of the dorsal brain including the region of the dorsal neurons (Fig. S1). In comparison, immunostaining with phospho-p38 MAPK antibody (hereafter also referred as p-p38) also showed clear labeling in the protocerebrum (Fig. 1F), but p-p38 staining of clock neurons was restricted to much fewer cells. We found reliable staining only in the DN_1a_s (Fig. 1F, G) and in one experiment also in the l-LN_v_s (not shown). This discrepancy might be due to the specificity of p-p38 antibody, which rather represents the current activation pattern than expression pattern of p38. Generally, tiny amounts of activated kinases are sufficient for effective signaling in transduction cascades. Thus, the amount of activated p38 might be well below the detection limit of the p-p38 antibody in the majority of clock neurons. In addition, p38 may be temporally phosphorylated as shown for the hamster SCN, where activated p38 was only high in the late day and early night [41]. Indeed, we noticed that p-p38 levels in the DN_1a_s as well as staining in the entire cortex of the brain depended also on the time of day and were only high towards the end of the night (Fig. 1D,E2 compared to Fig. 1F,G2).

To finally exclude any unspecific antibody labeling, antibody staining on two *p38* null mutants, *w^1118^;+;p38a^Δ1^* and *yw;p38b^Δ45^;+* (from now on referred to as *p38a^Δ1^* and *p38b^Δ45^*, respectively), was performed at ZT21 and p-p38 staining intensity was measured in DN_1a_s. Both p38 mutants displayed a significant reduction in phosphorylated p38 to 50% of wildtype level (p<0.05; Fig. S2). This clearly verifies the authenticity of the p-p38 antibody labeling, but also suggests the existence of both p38 isoforms in these cells.

Taken together, even if we did not get a complete overlap, *p38b*-driven GFP expression as well as p38 antibody staining indicates that both p38a and p38b are expressed in several clock neurons, most probably in the PDF-positive l-LN_v_s and s-LN_v_s as well as in the DN_1a_s. This finding coincides with other studies: Microarray studies on LN_v_s detected enriched *p38a* mRNA levels in the s-LN_v_s as compared to other brain regions [50]. Furthermore, Mef2, a transcription factor well recognized as a downstream target of p38 MAPK signaling in *Drosophila* muscle [27] and mammalian myocytes, lymphocytes and neurons [29], [51]–[53], was shown to localize in all subgroups of *Drosophila* clock neurons [54] indicating p38 MAPK signaling in these cells.

### Darkness and clock dependent phosphorylation of p38 MAPK in DN_1a_s

So far, *Drosophila* studies mainly focused on p38 MAPK expression over a longer period of time, especially with regard to development [26], [37], [55]. Since the observed changes in the amount of phosphorylated p38 in the DN_1a_s at ZT9 and ZT21 (Fig. 1D–G) might also point to daily oscillating gene expression, we examined mRNA levels of *p38a* and *p38b* in the course of a day.

**Figure 2:**
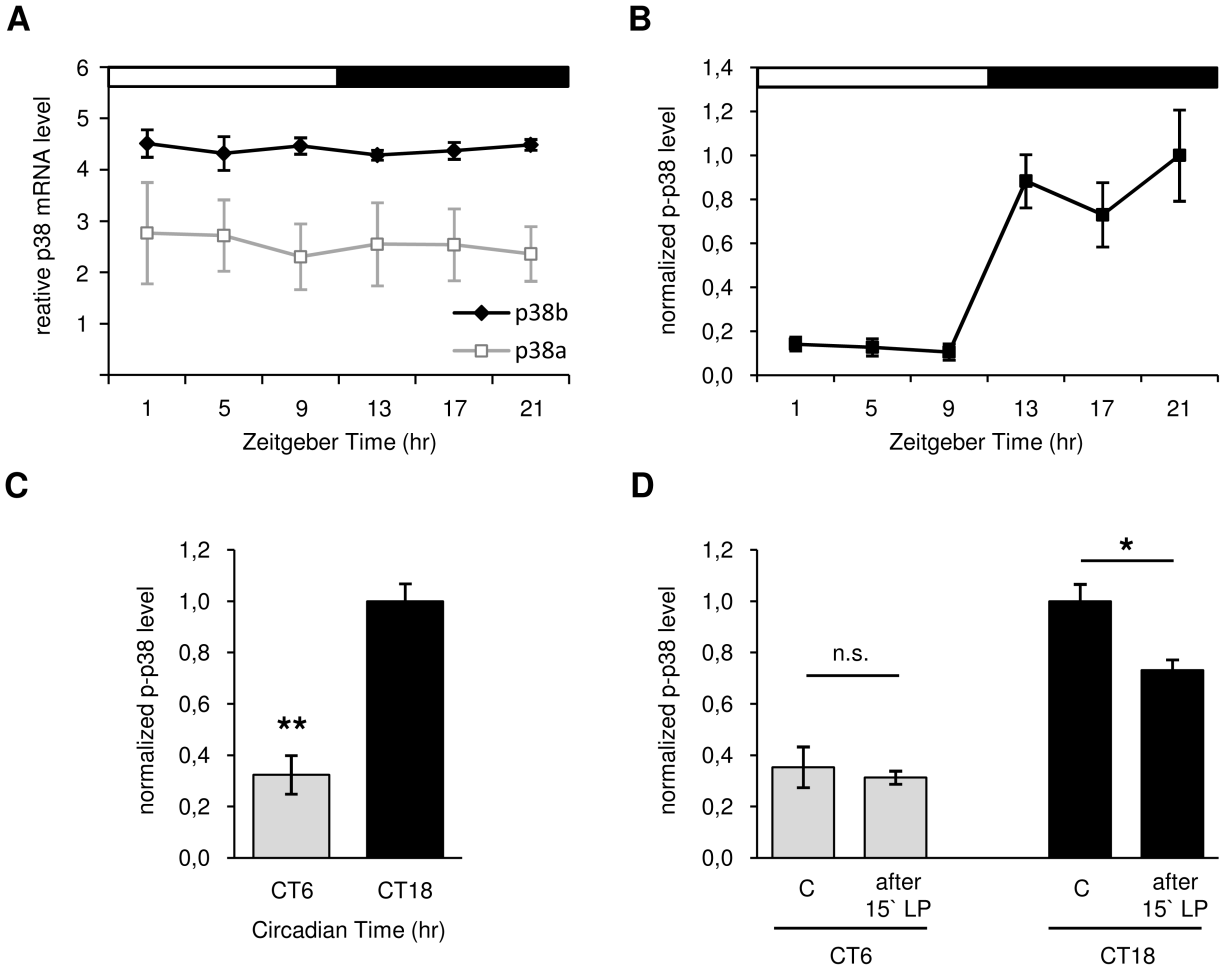
Daily p38 mRNA (A) and protein expression (B–D) in *Canton S* wildtype. A: Quantitative real-time PCR on head extracts revealed constant mRNA expression throughout the day with allover higher levels of *p38b* compared to *p38a* (p<0.001). B: Antibody staining with anti-p-p38 on adult brains displayed rhythmic phosphorylation of p38 in DN_1a_s in LD with significant higher p-p38 levels occurring during the night than in the day (p<0.05). C: A highly significant reduction of active p38 in DN_1a_s at CT6 compared to CT18 in DD indicates a clock-controlled activation of p38 (p<0.001) D: Only a 15 minute light pulse (LP) during subjective night (CT18) and not during the subjective day (CT6) leads to a reduction in active p38 in DN_1a_s, suggesting a clock-dependent photic reduction of active p38. The “C” in D indicates control brains without 15 minute light pulse (LP). Error bars show SEM. Significant differences (p<0.05) are indicated by *, highly significant differences (p<0.001) by **.

Quantitative real-time PCR (qPCR) from head extracts of *Canton S* wildtype flies revealed an allover higher expression level of *p38b* compared to *p38a* throughout the day (p<0.001; Fig. 2A). This is consistent with data published in a microarray-based atlas of gene expression in *Drosophila* (Flyatlas - http://www.flyatlas.org). Moreover, we did not discover any circadian oscillations of p38 isoforms on the transcriptional level, which is reminiscent on findings in fungi [44] and mammals [41], [56]. Very similar to our study, the latter papers demonstrated rhythmic phosphorylation of p38 throughout the day while total protein levels remained constant. This clearly indicates that activation and not expression of p38 is clock-controlled.

For studying oscillations in active p38 in more detail, immunohistochemistry on *Canton S* wildtype brains was carried out in LD 12∶12 at different times of day. Triple-labeling with anti-p-p38, anti-VRI and anti-PDF revealed daily oscillation in p38 phosphorylation in DN_1a_s, with low levels during the light phase (ZT1-9) and significantly higher levels in the dark (ZT13-21) (p<0.05; Fig. 2B). Furthermore, the average number of p-p38-positive DN_1a_s per hemisphere was significantly higher at night than during the day (p<0.05; Fig. S3). The diurnal oscillation in phosphorylated p38 in DN_1a_s strongly points to a clock-mediated activation of p38 within the circadian system. To test whether these diurnal variations in active p38 are indeed clock-controlled or just represent a direct response to darkness, p-p38 staining intensity in the DN_1a_s was measured under constant conditions at CT6 and CT18. Interestingly, similar to our observations in LD, the level of active p38 was significantly lower in the subjective day than subjective night confirming our hypothesis of a clock-controlled phosphorylation of p38 (p<0.001; Fig. 2C). Since previous studies in mice [57]–[59] and hamsters [41] also suggested a light dependent regulation of ERK and p38 activity in the SCN, we further exposed flies at CT6 and CT18 for 15 minutes to light and dissected brains before and after light pulse treatment. While levels of active p38 at CT 6 remained constant, light pulse at CT 18 led to a significant decrease in p-p38 signal (p<0.05; Fig. 2D). These results indicate an additional light-induced regulation (depression) of p38 activity.

Taken together, our findings are in strong favor of a clock-controlled phosphorylation of p38. Both *p38a* and *p38b* are constantly expressed throughout the day and display no circadian regulation on transcriptional level. Activation of p38 MAPK, however, seems to be clock-regulated, showing high levels of active p38 during the night and low levels during the day as we could show for the DN_1a_s. This would argue for a night-time specific function of p38 within the clock of these neurons. Nevertheless, we have to admit that the DN_1a_s are not the clock neurons that are most important for the control of behavioral rhythmicity. Future studies have to show, whether a cyclic activation of p38 does also occur in the s-LN_v_s.

### p38b knockdown and overexpression in clock neurons induce period lengthening

**Figure 3:**
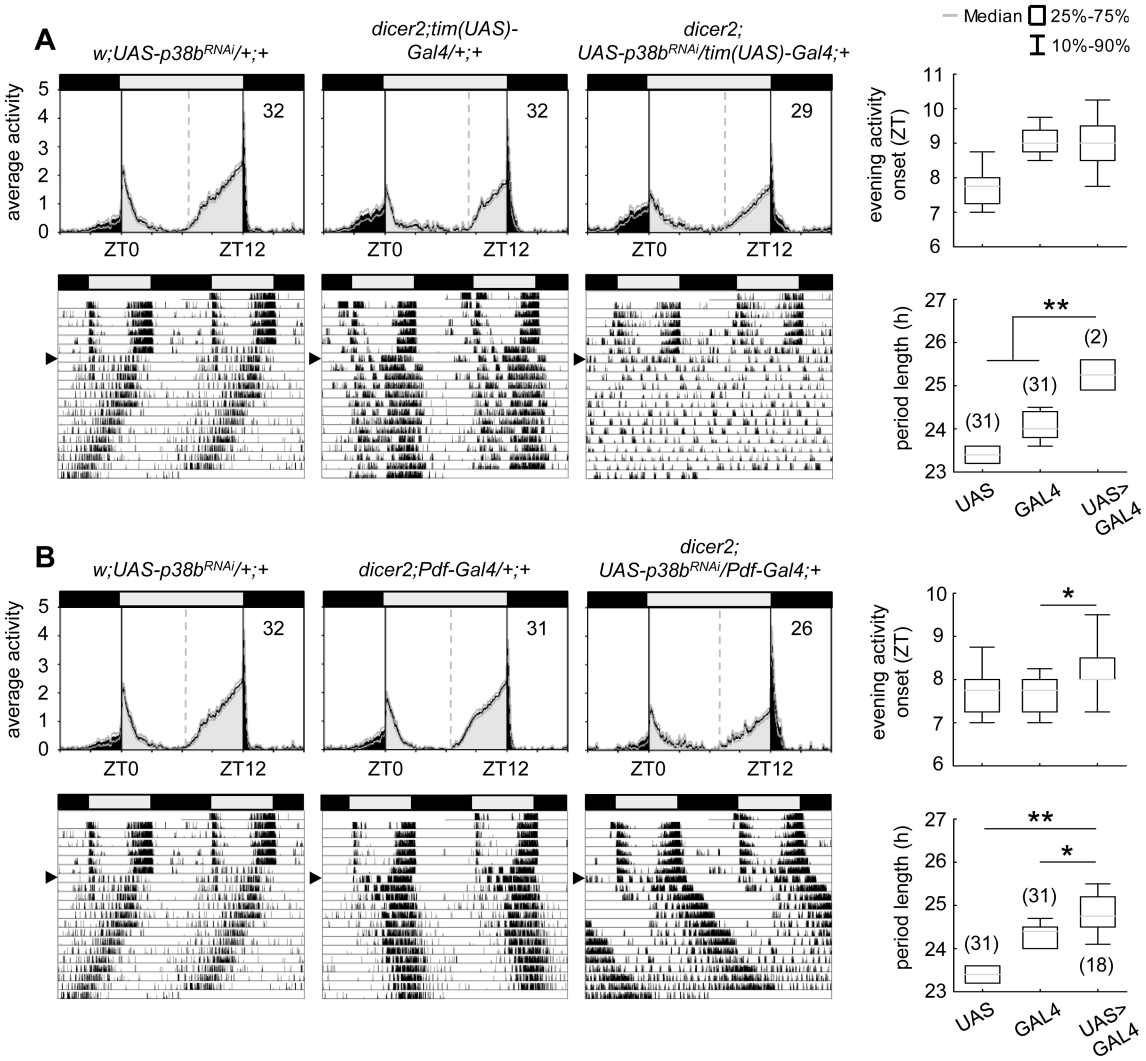
Locomotor activity rhythms of p38b knockdown flies with respective controls. Flies were recorded in LD 12∶12 for 6 days and subsequently in DD for at least 14 days. A daily average activity profile for day 2–7 in LD was calculated for each genotype and is shown above the double-plot of a representative actogram (left panels in A and B). In addition, for each genotype the onset of evening activity in LD (upper right panel in A and B) as well as the average free-running period in DD (lower right panel in A and B) was determined and is depicted as boxplot in the right panel. While average activity profiles of *dicer2;UAS-p38b^RNAi^/tim(UAS)-Gal4;+* (A) displayed wildtype-like behavior in LD with activity bouts around lights-on and lights-off, evening activity onset of *dicer2;UAS-p38b^RNAi^/Pdf-Gal4;+* flies (B) was significantly delayed compared to respective controls. When transferred to DD, *dicer2;UAS-p38b^RNAi^/tim(UAS)-Gal4;+* mainly became arrhythmic (see Table 1 and lower left panel in A), just 7% remained rhythmic displaying a significant longer free-running period than *UAS*- and *Gal4*-controls (lower right panel in A). p38b knockdown in PDF-expressing neurons also significantly lengthened the free-running period in DD as compared to respective controls (lower panels in B), and 58% of the flies remained rhythmic (see Table 1). Bars above the daily average activity profiles and actograms depict the light regime of the LD 12∶12 cycle and black arrowhead indicate the shift to constant DD. Black lines in daily average activity profiles represent mean relative activity, gray lines SEM and dotted grey lines the calculated evening activity onset. Gray lines in boxplots illustrate the median, boxes 25–75%, and whiskers 10–90% of the data. UAS refers to respective UAS-control, GAL4 to respective Gal4-control and UAS>GAL4 to the experimental line. Significant differences (p<0.05) are indicated by *, highly significant differences (p<0.001) by **. Numbers in brackets indicate *n*.

Locomotor activity recordings are a well-suited technique for investigating circadian behavioral rhythms in *Drosophila melanogaster*. When entrained to LD cycles wildtype flies display a typical bimodal activity pattern with an anticipatory morning and evening activity peak around lights-on and lights-off. In constant darkness this rhythmic locomotor behavior proceeds with its internal individual period reflecting the pace of the endogenous clock. To examine the role for p38 MAPK within the circadian system, we used transgenic RNA interference (RNAi) to reduce *p38b* RNA levels and thus p38b activity in different subsets of clock neurons, and screened for altered behavioral rhythms in LD as well as in constant dark conditions (DD). For RNAi-mediated p38b knockdown a *w;UAS-p38b^RNAi^;+* line was combined with different drivers as well as a *UAS-dicer2;+;+* line (*dicer2*). We first used *dicer2*;*tim(UAS)-Gal4;+*, a driver line with a broad expression pattern that allows ubiquitous expression in all clock cells. Daily activity patterns of *dicer2;UAS-p38b^RNAi^/tim(UAS)-Gal4;+* flies were similar to those of control flies showing normal wildtype LD behavior with activity peaks around lights-on and lights-off (Fig. 3A). To test the effectiveness of *p38b* transgenic RNAi, we performed qPCR on head extracts and found no significant reduction in *p38b* mRNA level in our experimental line. This may be due to a small number of p38b-positive clock neurons compared with the total number of p38b-expressing neurons within the brain (Fig. S1 compared to Fig. 1). Thus, *w;UAS-p38b^RNAi^;+* was additionally combined with *da-Gal4*, a line that expresses Gal4 in most tissues throughout development [60]. Using the broader driver, we finally observed a significant decrease in *p38b* mRNA level in *w;UAS-p38b^RNAi^/+;da-Gal4/+* compared to respective controls, confirming the effectiveness of our *p38b^RNAi^* construct (p<0.05; Fig. S4). Since we found no behavioral phenotype in LD, we next focused on locomotor behavior of *dicer2;UAS-p38b^RNAi^/tim(UAS)-Gal4;+* flies under constant conditions using χ^2^-periodogram analysis. Surprisingly, 93% of the flies were arrhythmic (Table 1) and only 7% showed rhythmic locomotor behavior with a prolonged free-running period of 25.3 h (p<0.001; Fig. 3A; Table 1). Considering the fact that besides clock neurons *dicer2;tim(UAS)-Gal4;+* additionally drives expression in glia cells, we wanted to rule out a glia-specific effect on rhythmicity and period length. Therefore, we restricted p38b knockdown solely to the PDF-expressing clock neurons, the s-LN_v_s and the l-LN_v_s, using the more specific clock driver *dicer2;Pdf-Gal4;+*. *Dicer2;UAS-p38b^RNAi^/Pdf-Gal4;+* flies showed a later onset of evening activity and a higher activity after lights-off than control flies in LD (Fig. 3B) as well as a significantly prolonged free-running period of 24.8 h in DD (p<0.05; Fig. 3B; Table 1). Only about half of the flies were arrhythmic as opposed to 93% of *dicer2;UAS-p38b^RNAi^/tim(UAS)-Gal4;+* flies (Table 1). These findings suggest that p38 has indeed a functional role within the circadian system and that its specific knockdown in the clock neurons mainly delays evening activity and lengthens the free-running period.

**Table 1:** Rhythmicity and period length of all investigated genotypes in constant darkness (DD) according to χ^2^-periodogram analysis.

Genotype	*n*	arrhythmic^1^ (%)	rhythmic (%)	Period (hr± SEM)	Power^2^ (%V ± SEM)
*w;UAS-p38b^RNAi^/+;+*	32	3	97	23.4±0.04	35.4±1.49
*dicer2;Pdf-Gal4/+;+*	31	0	100	24.3±0.05	38.5±1.84
*dicer2;tim(UAS)-Gal4/+;+*	32	3	97	24.1±0.06	38.5±1.91
*dicer2;UAS-p38b^RNAi^/Pdf-Gal4;+*	31	42	58	24.8±0.11	38.3±3.23
*dicer2;UAS-p38b^RNAi^/tim(UAS)-Gal4;+*	28	93	7	25.3±0.35	42.4±8.00
*w;UAS-p38b^KD3^/+;UAS-p38b^KD8^/+*	31	13	87	23.8±0.04	33.9±1.52
*w;Pdf-Gal4/+;+*	30	3	97	24.0±0.07	35.6±2.25
*w;tim(UAS)-Gal4/+;+*	32	0	100	24.1±0.04	40.1±2.05
*w;UAS-p38b^KD3^/Pdf-Gal4;UAS-p38b^KD8^/+*	32	6	94	25.1±0.07	35.5±1.48
*w;UAS-p38b^KD3^/tim(UAS)-Gal4; UAS-p38b^KD8^/+*	30	3	97	25.1±0.08	35.6±1.89
*UAS-p38b^DN-S^;+;+*	32	6	94	24.0±0.04	34.2±1.56
*UAS-p38b^DN-S^;Pdf-Gal4/+;+*	32	3	97	26.5±0.09	30.0±0.93
*UAS-p38b^DN-S^;tim(UAS)-Gal4/+;+*	30	3	97	25.9±0.06	34.1±1.93
*UAS-p38b^+^;+;+*	32	22	78	23.2±0.06	22.2±1.07
*UAS-p38b^+^;Pdf-Gal4/+;+*	31	0	100	25.3±0.04	35.5±1.85
*UAS-p38b^+^;tim(UAS)-Gal4/+;+*	29	0	100	25.5±0.06	41.7±2.24
*w;+;UAS-p38a^RNAi^/+*	32	0	100	23.8±0.05	44.7±2.57
*dicer2;Pdf-Gal4/+;UAS-p38a^RNAi^/+*	32	0	100	24.6±0.06	47.9±2.62
*dicer2;tim(UAS)-Gal4/+;UAS-p38a^RNAi^/+*	32	6	94	24.5±0.07	39.1±1.81
*dicer2;UAS-p38b^RNAi^/+;UAS-p38a^RNAi^/+*	32	12	88	23.9±0.05	26.3±1.37
*dicer2;UAS-p38b^RNAi^/tim(UAS)-Gal4; UAS-p38a^RNAi^/+*	27	41	59	24.9±0.08	26.3±1.99
*w^1118^*	32	10	90	23.3±0.07	24.5±1.05
*p38a^Δ1^*	29	47	53	23.2±0.10	23.7±1.35
*p38b^pex41^*	29	14	86	23.6±0.08	32.4±1.30
*p38b^Δ45^*	28	21	79	23.7±0.07	28.3±1.91

*n* indicates the number of tested flies per genotype that survived locomotor recordings. Power and period values were averaged over all rhythmic flies for each genotype.

1 Flies with power values <20 were defined as arrhythmic.

2 Power is a measure of rhythmicity and is given in % of variance.

**Figure 4:**
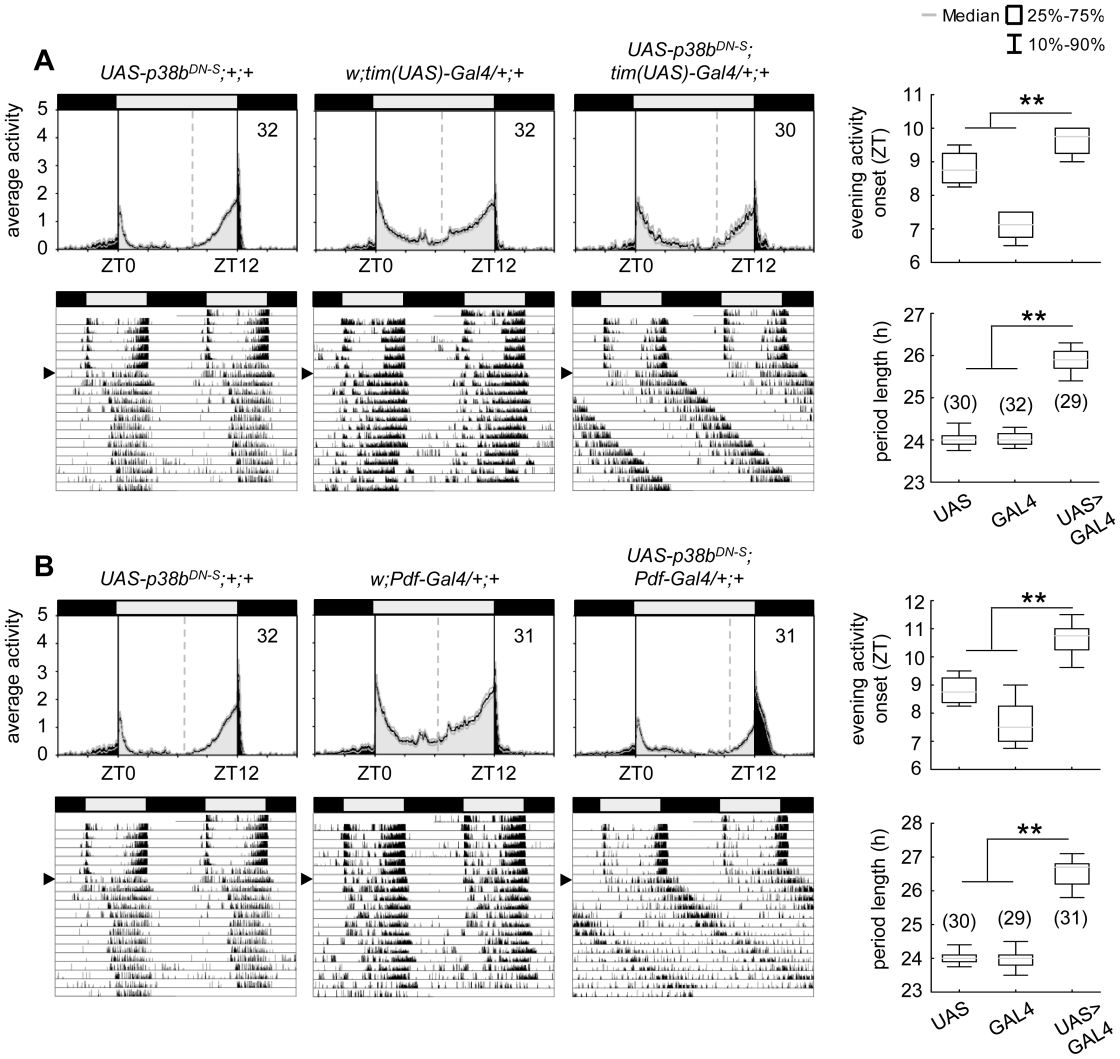
Locomotor activity rhythms of flies expressing a dominant-negative form of p38b *(p38b^DN-S^)* in *Drosophila* clock neurons and respective controls. Both, expression of a dominant negative form of p38b in either all clock neurons (*UAS-p38b^DN-S^;tim(UAS)-Gal4/+;+*) or just in a subset of clock cells, the PDF-positive LN_v_s (*UAS*-*p38b^DN-S^;Pdf-Gal4/+;+*), resulted in a diurnal activity profile with a significantly delayed evening activity onset in comparison with respective controls (upper panels in A and B). This delay in evening activity is accompanied by a significantly prolonged free-running period in *UAS*-*p38b^DN-S^;tim(UAS)-Gal4/+;+* (lower panels in A) as well as in *UAS*-*p38b^DN-S^;Pdf-Gal4/+;+* flies(lower panels in B), when released into constant darkness. For recording and processing of activity data as well as for figure labeling see Figure 3.

To further confirm our hypothesis of p38 functioning in the clock three additional constructs were expressed to interfere with endogenous p38b: two *UAS-p38b kinase-dead* transgenes (*UAS-p38b^KD3^* and *UAS-p38b^KD8^*) and a dominant-negative *UAS-p38b* transgene (*UAS-p38b^DN-S^*). Interestingly, simultaneous expression of *UAS-p38b^KD3^* and *UAS-p38b^KD8^* in either PDF- or TIM-expressing neurons resulted in a delayed onset of evening activity and a prolonged free-running period under DD compared to respective controls, but did not cause any arrhythmicity (p<0.001; Fig. S5; Table 1). This phenotype became even more obvious when the dominant-negative *p38b* transgene was expressed in all clock cells (using *w;tim(UAS)-Gal4;+*) or only in the LN_v_s (using *yw;Pdf-Gal4;+*): evening activity was delayed in LD and, in DD, free-running period was lengthened for about 2 h in *UAS-p38b^DNS^;tim(UAS)-Gal4/+;+* flies (p<0.001; Fig. 4A; Table 1) and for about 2.5 h in *UAS-p38b^DNS^;Pdf-Gal4/+;+* flies as compared to controls (p<0.001; Fig. 4B; Table 1). Again, no higher fraction of arrhythmic flies was observed (Table 1).

The high number of arrhythmic flies after knockdown of p38b, which was not observed with any of the other constructs, points to putative off-target effects of the *p38b^RNAi^* construct. Off-target effects can never be excluded with the RNAi technology and are more likely to occur if the RNAi construct is expressed in many neurons as was the case with the *tim-gal4* driver. When *p38b^RNAi^* was expressed with *pdf-gal4*, that drives in only 16 neurons in the brain, the number of arrhythmic flies was significantly lower (χ^2^-test: χ^2^ = 17.02; p<0.001). Thus, it is most likely that the *p38b^RNAi^* construct has off-target effects causing arrhythmicity in addition to its specific effects on clock speed. To test, whether the molecular clock is still running in DD after knockdown of p38b, we immunostained brains of *dicer2;UAS-p38b^RNAi^/Pdf-Gal4;+* flies with anti-PER and anti-TIM throughout the circadian cycle on day 4 in DD (Fig. S6). We found that the molecular cycling persisted, just the phase of the oscillation was delayed in comparison to controls, which is consistent with the long period of the flies. We conclude that p38 mainly affects the speed of the clock and has little if any effects on the stability and robustness of the molecular clock cycling.

Since *p38b^RNAi^* knockdown and expression of non-functional p38b cause period lengthening of locomotor free-running rhythms, we next wondered what would happen if we overexpress wildtype p38b (*UAS-p38b^+^*) in different subsets of clock neurons. Surprisingly, although p38b overexpression might have been expected to give the opposite effect of *p38b^RNAi^* (i.e., short locomotor free-running period), *UAS-p38b^+^;Pdf-Gal4/+;+* and *UAS-p38b^+^;tim(UAS)-Gal4/+;+* exhibited significantly later evening activity in LD and longer locomotor free-running rhythms in DD that were similar to those of *p38b^RNAi^* and *p38b^KD^* flies (p<0.001 and p<0.001 respectively; Fig. S7 and Table 1). This suggests that there is an optimal level of p38b for provoking locomotor activity rhythms with normal period.

Taken together our results indicate that wildtype levels of functional p38b are required for wildtype timing of evening activity and normal/wildtype free-running rhythms under constant conditions. Furthermore, already p38b knockdown or overexpression restricted to the LN_v_s (PDF-neurons) is sufficient to cause free-running rhythms with long period. This is well consistent with the dominant role of the s-LN_v_s, in which we found p38 expression, in controlling rhythms under constant darkness (reviewed in [61]). Since the oscillation speed was significantly affected by p38b manipulation, we rather assume a function of p38 MAPK in the core of the clock than in its input pathway.

### p38a knockdown recapitulates the p38b knockdown phenotype

**Figure 5:**
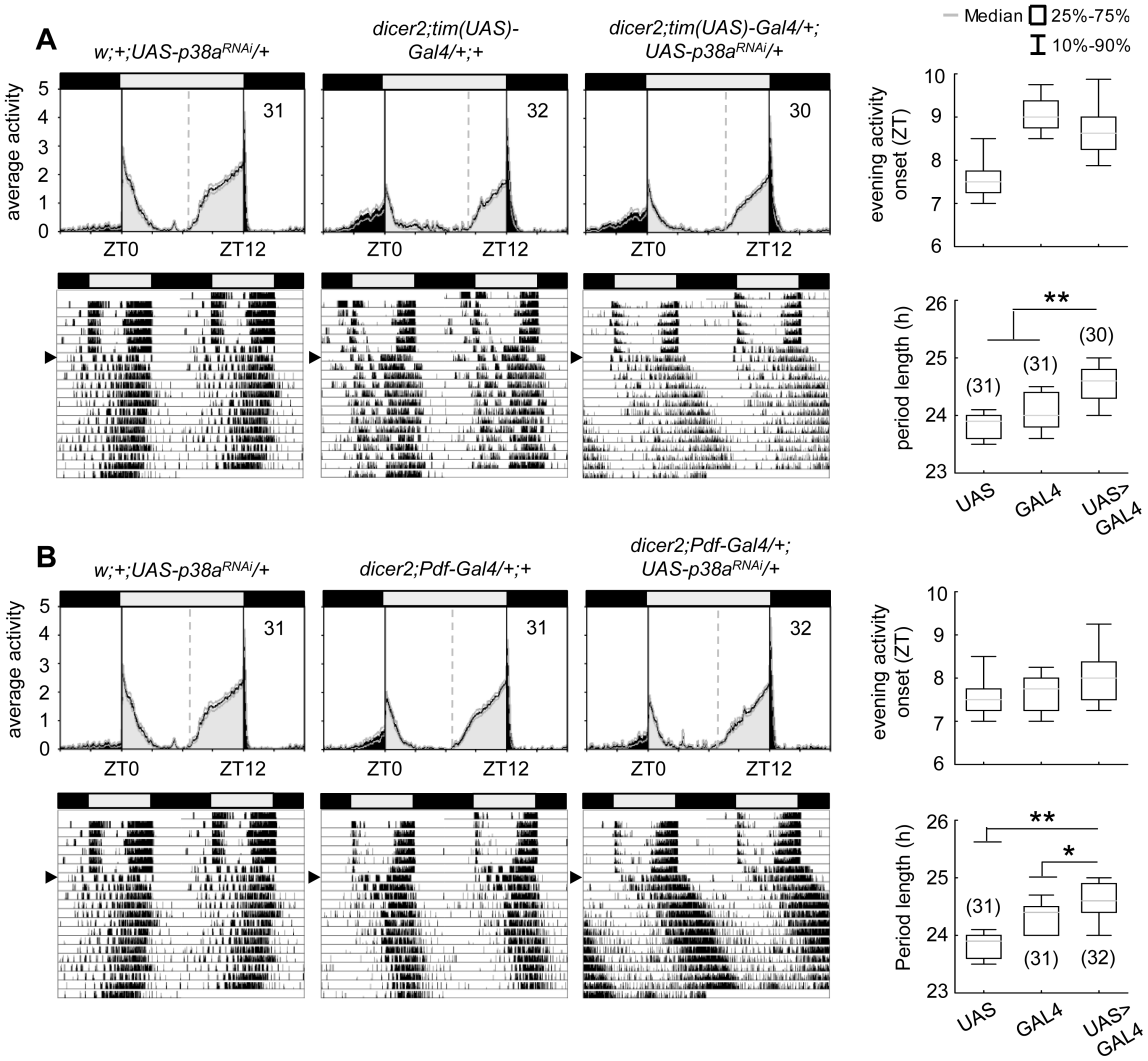
Locomotor activity rhythms of p38a knockdown flies and respective controls. Average activity profiles of *dicer2;UAS-p38b^RNAi^/tim(UAS)-Gal4;+* (upper panel in A) and *dicer2;UAS-p38b^RNAi^/Pdf-Gal4;+* (upper panel in B) displayed wildtype-like behavior in LD with activity bouts around lights-on and lights-off and did not differ from those of control flies. Evening activity onset was not delayed in the two mutant strains. However, when released into constant darkness both, p38a knockdown in TIM- (lower panels in A) and PDF-expressing neurons (lower panels in B) resulted in significant prolonged free-running rhythms in comparison to respective controls. For recording and processing of activity data as well as for figure labeling see Figure 3.

As shown before, the p38a isoform appeared to be co-expressed in the clock neurons (Fig. S2) raising the question whether p38a has also a possible function within the circadian system. To test this, we down-regulated p38a either in all clock cells (using *dicer2;tim(UAS)-Gal4;+*) or only in the LN_v_s (using *dicer2;Pdf-Gal4;+*), as done before for p38b. The effectiveness of *p38a* transgenic RNAi was successfully confirmed via qPCR on *w;+;UAS-p38a^RNAi^/da-Gal4* fly heads (p<0.001; Fig. S4). Down-regulation of p38a had generally weaker effects than down-regulation of p38b: it did not significantly delay evening activity in LD and, in DD, period was not lengthened as dramatically as after manipulation of p38b protein level (Fig. 5). Nevertheless, χ^2^-periodogram analysis revealed that both experimental lines (*dicer2;tim(UAS)-Gal4/+;UAS-p38a^RNAi^/+* and *dicer2;Pdf-Gal4/+;UAS-p38a^RNAi^/+*) had significantly longer free-running periods than the respective controls (p<0.001 and p<0.05 respectively; Fig. 5A,B; Table 1). This result strongly argues for a clock-related role for p38a besides p38b.

### Complete loss of either p38b or p38a does not disturb circadian rhythms

After we found that down-regulation of p38b or p38a significantly affected the flies' free-running rhythms, we aimed to test whether a complete loss of either isoform does affect rhythmicity in a similar way. To our surprise this was not the case.

**Figure 6:**
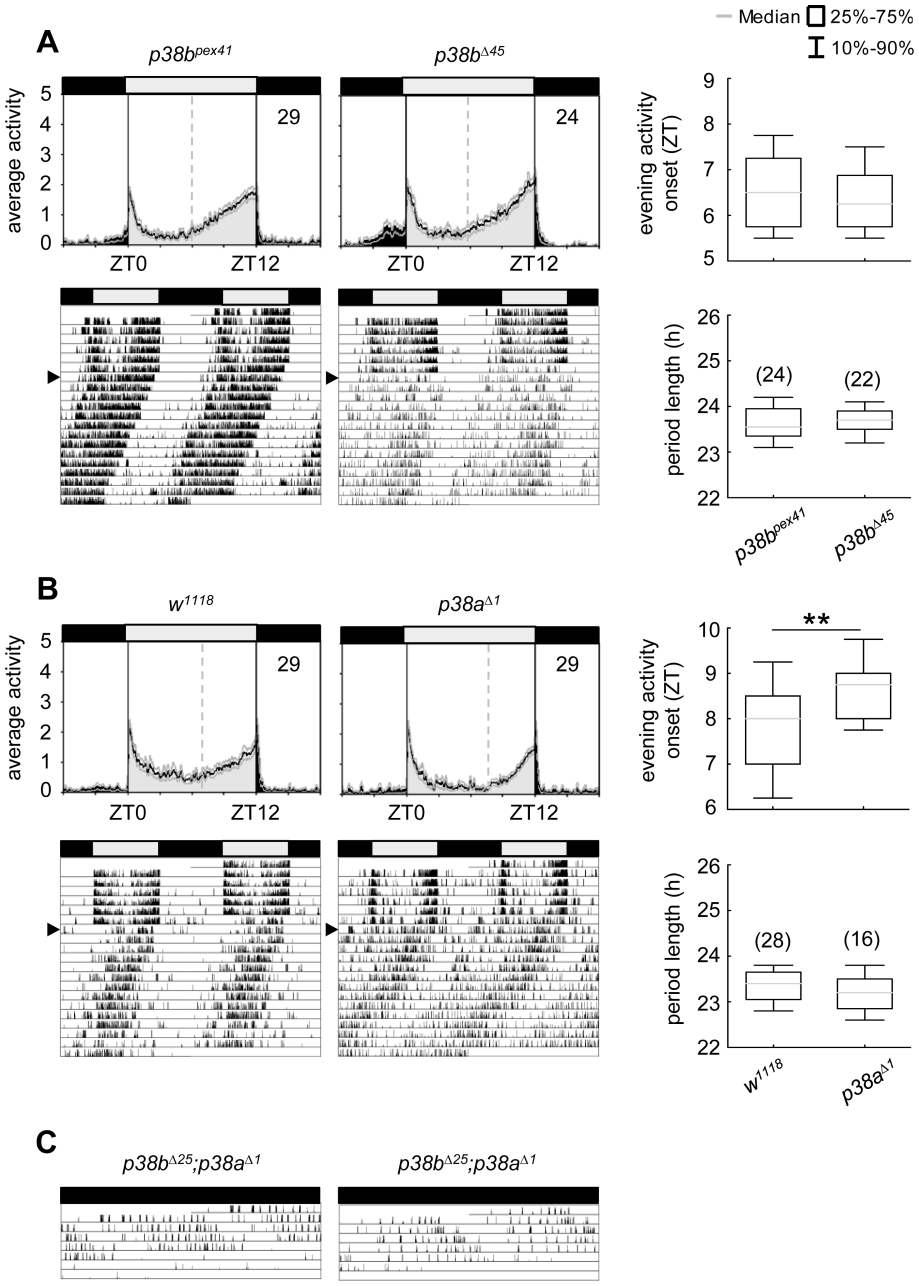
Locomotor activity rhythms of *p38b* and *p38a* null mutants and hypomorphic double mutant flies. Both *p38* null mutants, *p38b^Δ45^* (upper panels in A) and *p38a^Δ1^* (upper panels in B), displayed wildtype-like behavior with activity bouts around lights-on and lights-off when recorded in LD 12∶12. Even if evening activity onset of *p38a^Δ1^*seems to be delayed compared to *w^1118^*, this delay did not result in a longer free-running period under constant darkness (lower panels in B). Similarly, flies, lacking the *p38b* gene, also showed comparable free-running rhythms as their respective controls (lower panels in A). Activity data in C show two representative single actograms of a double mutant strain with a hypomorphic *p38b* allele (*p38b^Δ25^;p38a^Δ1^*). Since these flies are hardly viable and die within 3–6 days after emergence of the pupa, flies were already entrained to LD12∶12 during pupal stage and subsequently monitored in DD conditions after eclosion. Even if periodogram analysis was not possible due to the short recording period, *p38b^Δ25^;p38a^Δ1^* flies clearly showed a long free-running period when kept in constant darkness (C). For recording and processing of activity data as well as for figure labeling see Figure 3.

Although *p38b^Δ45^*, a *p38b* null mutant, displayed a slightly enhanced percentage of arrhythmic flies in DD, this was also the case for its precise excision line *p38b^pex41^*, indicating some background effect on rhythmicity (Table 1). Additionally, both lines showed similar activity profiles under LD conditions and did not differ in their free-running period (Fig. 6A; Table 1). Even if *p38a* null mutants, *p38a^Δ1^*, showed a later onset of evening activity and a higher fraction of arrhythmic flies than the *w^1118^* controls (Fig. 6B and Table 1), the free-running period of the rhythmic flies was not different from that of the controls. The higher percentage of arrhythmic flies might be associated with the prominent role of p38a in immune stress response [39]–[40], inflammation [26], [62] and lifespan [27]. Therefore flies lacking this isoform might be less healthy and display disrupted behavioral rhythms.

Our results suggest that the two isoforms might replace each other under certain conditions. According to Han et al. [26] p38a and p38b appear to have partial functional redundancy, because both isoforms are similarly activated in response to stress-inducing or inflammatory stimuli in cell culture experiments. This compensatory mechanism may be vital for the flies, when one of the two isoforms lacks completely. But the compensatory mechanism may be elusive when one isoform is only down-regulated in specific neurons that are not necessary for survival (e.g. the clock neurons): Lengthened free-running rhythms in DD just occurred, when either p38b or p38a levels in clock neurons were reduced, but not completely absent from the entire fly. We therefore suppose that either isoform overtakes the clock specific function of the other one only in its complete absence. As *p38a* mRNA levels were not increased in *p38b^Δ45^* flies and *p38b* mRNA levels were not elevated in *p38a^Δ1^* (Fig. S8), normal wildtype p38a or p38b levels seem to be sufficient to drive circadian rhythms in complete absence of the other isoform.

If our hypothesis is true, *p38b^Δ45^;p38a^Δ1^* double mutants should show long free-running periods. Unfortunately, the combination of both null alleles turned out to be lethal. Similarly, double mutants lacking the *p38a* gene and carrying a hypomorphic *p38b* allele (*p38b^Δ25^;p38a^Δ1^*) were hardly viable. Furthermore, flies that hatched had a very short life-span dying 3–6 days after emergence of the pupa, making it hard to investigate their free-running rhythms. Nevertheless, we were able to record the locomotor activity of two *p38b^Δ25^;p38a^Δ1^* mutants for 5–6 days, which were entrained to LD 12∶12 during pupal stage and immediately transferred into DD after eclosion (Fig. 6C). These flies free-ran with a long period until they died. In addition, we could simultaneously down-regulate p38a and p38b in TIM-positive neurons (*dicer2;UAS-p38b^RNAi^/tim(UAS)-Gal4;UAS-p38a^RNAi^/+*). Such flies exhibited also significant longer free-running periods in DD than the relevant controls (p<0.001; Table 1).Together, our findings strongly indicate that both p38 isoforms are involved in the control of locomotor activity rhythms under constant conditions and that they can partly replace each other.

### Expression of the dominant negative form of p38b phase delays the molecular circadian clock

**Figure 7:**
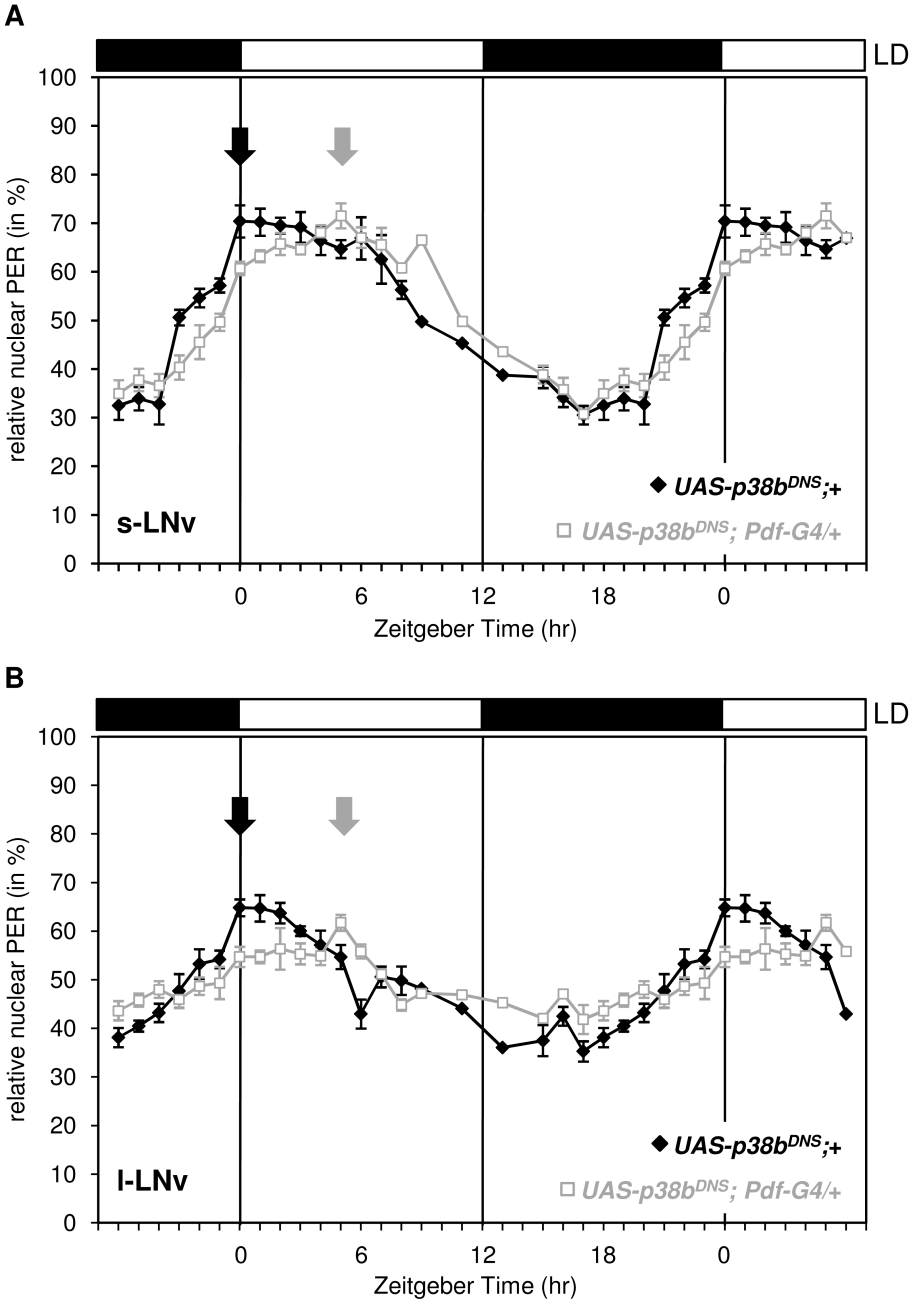
Daily oscillations of nuclear PER in s-LN_v_s and l-LN_v_s of flies expressing a dominant negative form of p38b in these cells. Flies were entrained in LD 12∶12, dissected every one to two hours and staining intensity of nucleus and cell body was measured as described in Material and Methods. Nuclear PER staining intensities were normalized to total staining and tested for statistically significance. Expression of the dominant negative form of p38b phase delayed nuclear accumulation of PER in the s-LN_v_s (A) and l-LN_v_s (B). Arrows indicate the maxima of nuclear PER staining that occurred significantly later in *UAS-p38b^DN-S^;Pdf-Gal4/+;+* flies than in control flies. This delay in nuclear PER accumulation in PDF-positive clock neurons is well consistent with the shifted evening activity in these flies. Bars above the graphs depict the light regime of the LD 12∶12.

Delayed evening activity and long free-running rhythms are often associated with a delayed nuclear entry of PER and TIM, an event in the molecular cycle which is mainly regulated via phosphorylation of PER by proline-directed kinases and SGG [12] as well as of TIM by SGG [63]. To see whether the nuclear entry of PER is affected by p38 MAPK, we immunostained respective controls and flies, in which the dominant-negative form of p38b was expressed in the LN_v_s (*UAS-p38b^DN-S^;Pdf-Gal4/+;+*), in 1-hour intervals in LD and quantified the amount of nuclear PER in the s-LN_v_s and l-LN_v_s (Fig. 7). We chose *UAS-p38b^DN-S^;Pdf-Gal4/+;+* since the delay in evening activity under LD conditions was most prominent compared to other p38 mutant strains. Interestingly, we found a significant delay of nuclear entry of PER in both types of clock neurons that perfectly matched the delayed evening activity.

### p38b affects the degree of PER phosphorylation

There are several ways, how p38 could influence the phosphorylation degree of PER and this way influence the efficiency and speed of nuclear translocation: p38 may directly phosphorylate PER or it may activate or inhibit already known key kinases of PER. Accordingly, p38 was shown to phosphorylate and activate CK2 [34]–[35] making it possible that p38 lengthens the period of the molecular oscillations via CK2 finally leading to a delayed nuclear translocation of the PER-TIM heterodimer. Alternatively or in addition, p38 may work on phosphatases that reduce phosphorylation. Previous studies revealed that both, p38 [64] and CK2 [65], stimulate the activity of the protein phosphatase 2A (PP2A) in mammalian fibroblasts. PP2A on the other hand was shown to dephosphorylate and stabilize PER, thereby promoting PER's nuclear translocation in *Drosophila* clock cells [66]. Consequently, reduction of PP2A activity resulted in long free-running periods, the same phenotype we observed after manipulation of p38 levels.

**Figure 8:**
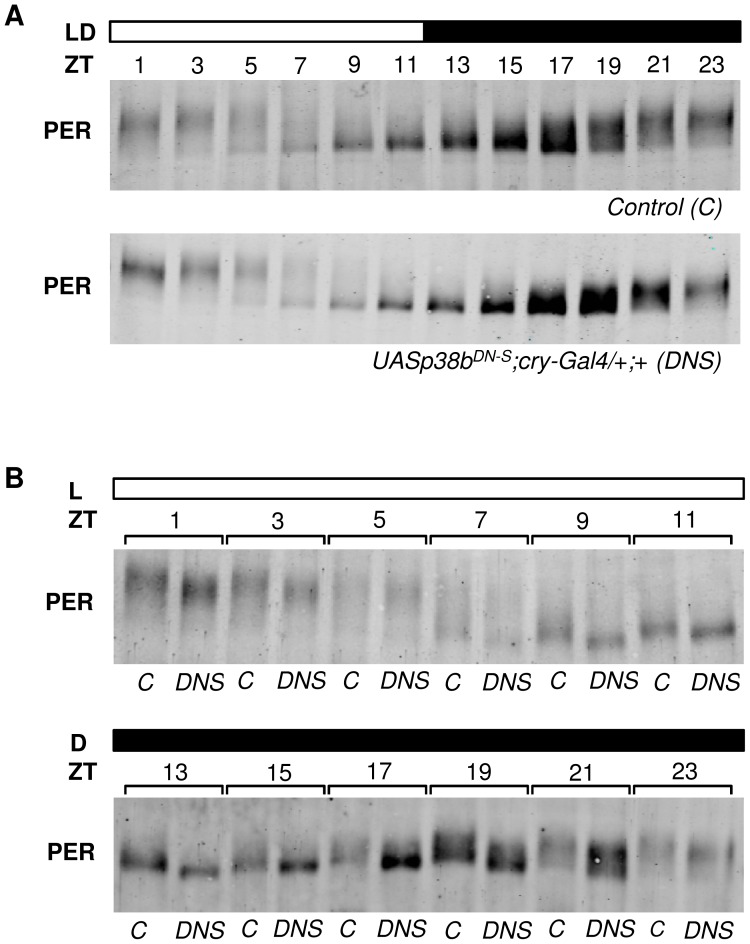
p38b promotes PER phosphorylation during the dark phase. To analyze daily phosphorylation of PER in flies that express the dominant-negative form of p38b in clock neurons and photoreceptor cells, we performed Western blots on head extracts after 4 days entrainment to LD 12∶12 cycles. According to our behavioral data, timing of PER accumulation was not affected in experimental flies (*UAS-p38b^DN-S^;cry-Gal4/+;+*) in comparison with their respective control (A). However, regarding the degree of PER phosphorylation we observed differences at all time points when we compared both genotypes. For better comparison Western blots were repeated and samples of control and *UAS-p38b^DN-S^;cry-Gal4/+;+* flies were plotted side by side for each ZT (B). Interestingly, flies with impaired p38 signaling indeed had less phosphorylated PER, showing the largest differences to the controls at the end of the night. Western blots were repeated 4 times and always gave similar results. Bars above the blots depict the light regime of the LD 12∶12. The “*C*” refers to respective control, *DNS* to *UAS-p38b^DN-S^;cry-Gal4/+;+*.

To test whether p38b affects the degree of PER phosphorylation, we performed Western Blots on head extracts of flies, in which the dominant-negative *p38b* transgene (*UAS-p38b^DN-S^*) was driven in all clock cells including the photoreceptor cells (in LD 12∶12). This time, we did not use *Pdf-gal4*, since Western Blots mainly reflect PER oscillations of the compound eyes (the oscillations of the 150 PER-expressing clock neurons can barely be seen behind the oscillations of the ∼1600 PER-expressing photoreceptor cells). Indeed, PER seemed to be less phosphorylated in flies with impaired p38b signaling (Fig. 8A). For a better comparison we repeated the Westerns blotting control and experimental flies for each ZT side by side (Fig. 8B). We found that PER was clearly less phosphorylated in the flies with impaired p38b signaling at all time points. This was most evident during the night being well consistent with the postulated high activity of p38 MAPK during darkness. We conclude that p38 promotes PER phosphorylation during the night. The lack of this phosphorylation may delay nuclear entry of PER during constant darkness and in this way lengthen the free-running period of the clock significantly.

### p38b phosphorylates PER *in vitro*

**Figure 9:**
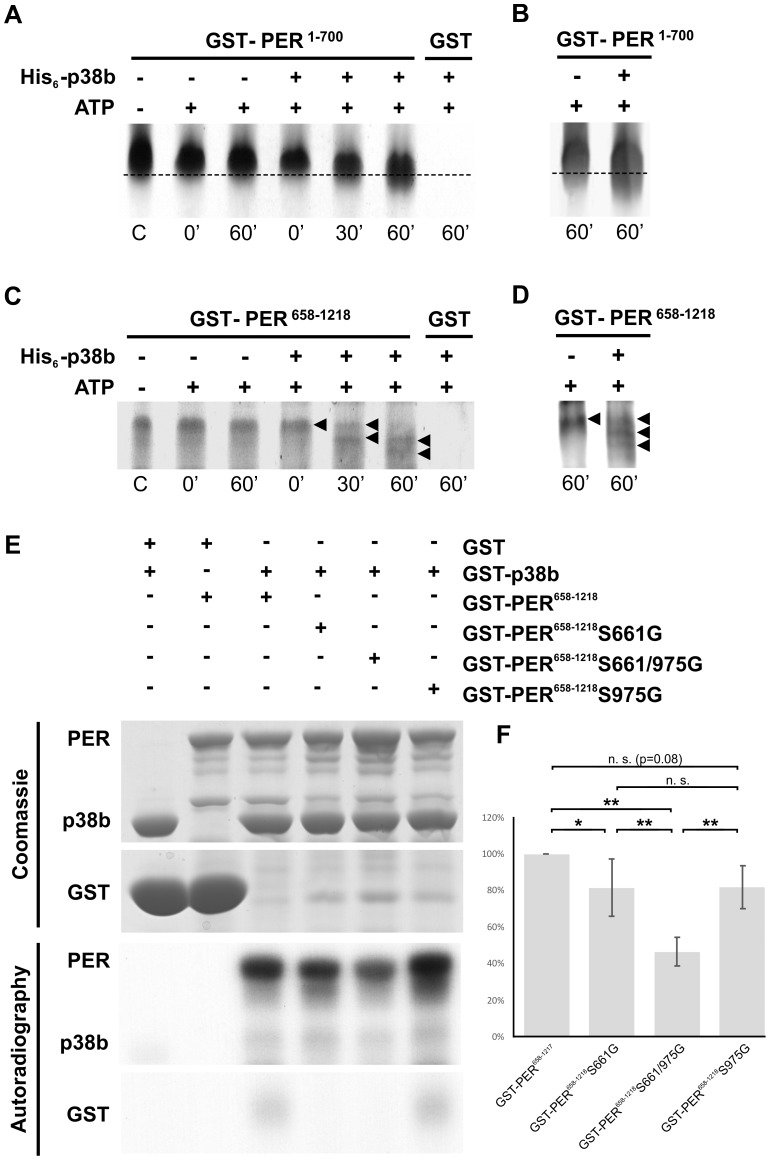
p38b phosphorylates PER *in vitro*. To test whether p38b phosphorylates PER *in vitro*, either non-radioactive kinase assays followed by urea-PAGE (A–D) or radioactive kinase assays with autoradiography (E–F) were performed. A–D: Non-radioactive kinase assays were conducted with poly-histidine tagged p38b (His_6_-p38b) and two truncated GST-tagged PER isoforms, GST-PER^1–700^ (A,B) and GST-PER^658–1218^ (C,D). Samples were subsequently separated with urea-PAGE and visualized by Coomassie staining (A,C). To further confirm PER's position in the gel two samples of the same gel were additionally blotted onto nitrocellulose membrane and immunolabeled using an anti-PER antibody and a secondary fluorescent antibody (B,D). While GST-PER^1–700^ without kinase did not shift within 60 minutes, the addition of His_6_-p38b induced a downward shift of GST-PER^1–700^ indicating phosphorylation of PER (A; dotted line). Immunoblots with anti-PER further confirmed the size as well as the shift of the GST-PER^1–700^ band (B). In addition to GST-PER^1–700^, GST-PER^658–1218^ also displayed band shifts after incubation with His_6_-p38b (C). This was most prominent after 60 minutes, when addition of His_6_-p38b resulted in two distinct shifted bands (black arrowheads), which could be additionally confirmed by Western blots (D). Time scale below graphs represents minutes after addition of His_6_-p38b, the “C” refers to control and represents substrate samples without kinase and ATP. (E) Radioactive *in vitro* kinase assays were conducted with the indicated GST-PER fusion proteins and GST-p38b. Control reactions were performed in the absence of GST-p38b or with GST in combination with GST-p38b. Coomassie staining proved loading of the indicated protein combinations. Below, phosphorylation of GST-PER proteins was detected by autoradiography. (F) For quantitative analysis five independent *in vitro* kinase assay experiments were performed and analyzed. For each reaction within a single experiment, autoradiography signal intensities were normalized to the corresponding Coomassie stained protein band. Values in the graph are shown as percentages of GST-PER^658–1218^ phosphorylation (100%; * p<0.05, ** p<0.005).

The next question to ask was, whether p38 can phosphorylate PER directly. PER becomes phosphorylated at multiple sites, some of which could be identified as predicted MAPK target sites [14]. Furthermore, Nemo/NLK, an evolutionarily conserved MAPK-related kinase, was shown to function as a priming kinase phosphorylating PER at the recently identified per-short phospho clusters and thereby stimulating phosphorylation of PER by DBT at several nearby sites [11], [13]. In addition, Ko et al. [12] could show that phosphorylation of serine 661 (Ser661) is a key phospho-signal on PER regulating the timing of PER's nuclear accumulation and that this phosphorylation event can be performed by proline-directed kinase(s), as could be shown for ERK *in vitro*. Mutant flies with blocked S661 phosphorylation site, display a delay in PER's nuclear entry in pacemaker neurons as well as long behavioral rhythms. Moreover, abolishing phosphorylation at Ser661 also diminishes the extent of hyperphosphorylation of PER *in vivo*, suggesting that the phosphorylated state of Ser661 regulates phosphorylation at other sites on PER. With Ser657 the authors also identified a phosphorylation target site of SGG, which seems to be phosphorylated in a manner dependent on priming at Ser661. Due to the similar phenotypes on molecular as well as behavioral level of *period* mutants lacking the phosphorylation site at Ser661 and *p38* mutants, we aimed to test whether p38 might also phosphorylate PER. Therefore, we created hexa-histidine tagged p38b (His_6_-p38b) and two GST tagged, truncated PER isoforms carrying GST amino-terminal fused either to amino acids 1–700 (GST-PER^1–700^) or to amino acids 658–1218 (GST-PER^658–1218^), and performed *in vitro* kinase assays. For visualization of protein phosphorylation, samples were subsequently separated on 9% urea-polyacrylamide gels followed by Coomassie staining. We chose urea-PAGE for protein separation, since urea does not mask the charge of the protein and therefore leads to longer runs of phosphorylated proteins due to the negative charges of phosphate residues (Fig. 9A, C). As we wanted to confirm the PER signal on the Coomassie gel, two samples of the gel were additionally blotted to nitrocellulose membrane following gel electrophoresis and detected by immunolabeling (Fig. 9B, D). Both, GST-PER^1–700^ and GST-PER^658–1218^, displayed obvious band shifts after 60 minutes of incubation with His_6_-p38b, while substrate controls without kinase did not shift in the appropriate time (Fig. 9A–D). Even if shifts are not extensive they are clearly visible. The appearance of several shifted bands (Fig. 9C, D) indicates that p38 might phosphorylate PER at several sites and thereby prime it for further phosphorylation. This could explain the overall amount of less phosphorylated PER we found in flies with impaired p38b signaling. Indeed, sequence analysis revealed two putative p38 consensus phosphorylation sites (PXS*P) in PER (Fig. S9): Ser661, that was shown to be phosphorylated by a proline-directed kinase and led to long free-running rhythms when mutated [12], and Ser975. To test whether p38 MAPK, which also belongs to the family of proline-directed kinases, phosphorylates PER at one of these sites, we mutated GST-PER^658–1218^ by replacing Ser with Gly either at position 661 (S661G), or at position 975 (S975G) or at both positions (S661G/S975G). Radioactive *in vitro* kinase assays were performed with bacterially expressed and purified GST-p38b together with wild-type and mutant forms of PER as substrates (Fig. 9E, F). Although p38b phosphorylated all forms of PER, phosphorylation was significantly reduced in the two single mutants and even further reduced in the S661/975G double mutant. Thus, we conclude that p38b can phosphorylate PER at S661 and at S975 - at least *in vitro*. Future studies have to show whether p38 does phosphorylate PER also *in vivo* at both sites and whether p38 may compete with other kinases at S661 (e.g. Nemo/NLK, ERK). The complex behavioral phenotypes (period-lengthening after down-regulation and overexpression of p38, as well as no effects of null mutations in p38a and p38b) argue for the putative interaction of several kinases in PER phosphorylation at S661.

In summary, our results demonstrate direct effects of p38 on circadian rhythms in behavior as well as on the molecular clock. Besides affecting the phosphorylation degree and nuclear entry of PER, p38 may influence the clock machinery in several ways due to its many putative targets in *Drosophila's* clock neurons. As we show here, one of the major p38 targets may be PER itself. Altogether, this places p38 in the center of multiple pathways that can affect circadian rhythms. Regarding its known role in transmitting cellular stress responses, p38 MAPK may even act as a factor that integrates responses of the circadian clock and the acute stress system to external stimuli. However, future studies have to reveal the exciting connection between the two systems in more detail.

## Materials and Methods

### Fly strains and constructs

Flies were raised on a standard cornmeal/agar medium at 25°C in LD 12∶12. To investigate locomotor activity in p38 mutant flies, we recorded two p38 knockout strains: *w^1118^;+;p38a^Δ1^* and *yw*;*p38b^Δ45^;+*(kindly provided by R. Cagan and A. Vrailas-Mortimer). The latter carries a 1065bp deletion in the coding region of *p38b*, while *w^1118^;+;p38a^Δ1^* flies completely lack the *p38a* locus. In addition the precise excision line *yw;p38b^pex41^;+* (a gift of A. Vrailas-Mortimer) served as control for *yw*;*p38b^Δ45^;+*. *w^1118^* flies served as control for the *w^1118^;+;p38a^Δ1^* mutants. Two double mutant strains, *p38b^Δ45^;p38a^Δ1^* and *p38b^Δ25^;p38a^Δ1^* (both provided by A. Vrailas-Mortimer; the latter exhibits a hypomorphic *p38b* allele) were used to knockout both p38 isoforms; but these turned out to be either lethal or only weakly viable in our hands. Therefore, we could not perform any statistical analysis of their locomotor activity rhythms. For studying p38 knockdown exclusively within the circadian clock, we used two different RNAi lines, *w;+;UAS-p38a^RNAi^* (Vienna Drosophila RNAi Center; #52277) and *w;UAS-p38b^RNAi^;+* (Vienna *Drosophila* RNAi Center; #108099), as well as a combination of both (*w;UAS-p38b^RNAi^;UAS-p38a^RNAi^*). To restrict RNAi-mediated gene silencing to specific subsets of clock neurons, RNAi lines were crossed to a *w;tim(UAS)Gal4;+* (kindly provided by Michael W. Young) as well as a *yw;Pdf-Gal4;+* driver line (kindly provided by Jeffrey C. Hall) and combined with a *UAS-dicer2;+;+* line (Vienna *Drosophila* RNAi Center; #60012) to further strengthen RNAi knockdown. In addition *yw;Pdf-Gal4;+* and *w;tim(UAS)-Gal4;+* flies were used to specifically overexpress wildtype p38b (*UASp38b^+^* kindly provided by T. Adachi-Yamada) as well as two non-functional p38b isoforms: a dominant-negative *UAS-p38b* transgene, *UAS-p38b^DN-S^* (donated by T. Adachi-Yamada), and an *UAS-p38b kinase-dead* transgene, *UAS-p38b^KD^*(a gift of A. Vrailas-Mortimer). The dominant-negative *p38b* allele was generated by replacing the Thr184 of the MAPKK target site with Ala leading to a complete loss of enzymatic activity [28]. The *UASp38b^KD^* transgenic line, however, was made by exchanging a Lys residue at 53 in the catalytic domain with Arg [27]. This single amino acid substitution still allows target binding, but blocks kinase activity (A. Vrailas-Mortimer, personal communication). Here we used flies with two *UAS-p38b^KD^* transgenes, a weaker *UAS-p38b^KD3^* and a stronger *UAS-p38b^KD8^* (*UAS-p38b^KD3^/CyO-GFP;UAS-p38b^KD8^/TM3Ser-GFP*). For studying the expression pattern of p38 within the brain of *Drosophila melanogaster* a *Canton S* (*CS*) wildtype strain was chosen as wildtype control for immunohistochemistry. In addition a *p38b-Gal4* enhancer trap line (kindly provided by A. Vrailas-Mortimer) was used in combination with *w;+;UAS-GFP^S65T^* (Bloomington Stock Center, #1522; donated by Karl Fischbach) to express green fluorescent protein (GFP) in the p38b-neurons revealing p38b expression in detail. To analyze PER cycling on Western Blots, we used a *w;cry-Gal4;+* driver line (kindly provided by F. Rouyer) to impair p38b signaling in *p38b^DN-S^*-flies.

For *in vitro* kinase assays, N-terminal hexa-histidine or GST-tagged p38b fusion proteins (His_6_-p38b and GST-p38b) were created by first PCR amplifying the full-length *p38b* open reading frame (ORF) using the cDNA clone as template and following primers: 5′-CCGATCGAAATGTCGCGCAAAATGGCCAAATTC-3′ and 5′-GGCGGCCGCGATTACTGCTCTTTGGGCAGGAGCTCA-3′. After digestion with PvuI and NotI, the PCR product was inserted into the multiple cloning site of E. coli expression vector pH6HTN His_6_HaloTag T7 (Promega) and further subcloned as an EcoRI/NotI fragment into the pGEX 4T3 vector (GE Life Sciences). In order to generate recombinant GST-PER fusion construct, two truncated sequences of *per*, either encoding amino acids 1–700 (PER^1–700^) or amino acids 658–1218 (PER^658–1218^), were subcloned into the pGEX 6P vector (GE Life Sciences). All constructs were confirmed by DNA sequencing before use.

GST-PER^658–1218^S661G (*pGEX6P-per^S661G^*) and GST-Per^658–1218^S975G (*pGEX6P-per^S975G^*) constructs were generated by mutagenesis PCR using *pGEX6P-per^658–1218^* as template. The primers 5′-CTCGTGGACGGGACCCATGGGCCCACTGGCGCCACTG-3′ and 5′-CAGTGGCGCCAGTGGGCCCATGGGTCCCGTCCACGAG-3′ were used to generate GST-*pGEX6P-per^S661G^* and the primers 5′-CTTGACGCCCACCGGGCCCACGCGCTCTCC-3′ and 5′GGAGAGCGCGTGGGCCCGGTGGGCGTCAAG-3′ were used for *pGEX6P-per^S975G^* generation. To generate the double mutant GST-PER^658–1218^S661/975G we performed a second mutagenesis PCR using *pGEX6P-per^S661G^* as template and the *pGEX6P-per^S975G^* mutagenesis primers as described above.

### Behavioral analysis

Locomotor activity of individual flies was recorded using the *Drosophila* Activity Monitoring (DAM) System (Trikinetics) as previously described [67]. Briefly, to investigate locomotor behavior 3–7 day old male flies were monitored in LD 12∶12 for 7 days (with a light intensity of 100 lux in the light phase) followed by additional 14 days in constant darkness (DD). In case of *p38b^Δ25^;p38a^Δ1^*, flies were entrained in LD 12∶12 during pupal stage and monitored directly after eclosion in DD conditions. All recordings took place under constant 20°C in a climate–controlled chamber. Raw data of individual light beam crosses were collected in 1-minute bins and displayed as double-plotted actograms using ActogramJ [68], a freely available Java plug-in of ImageJ (freely available at http://rsb.info.nih.gov/ij/). We generally excluded data of the first experimental day from analysis to exclude side effects of fly handling. For generating average daily activity profiles for single genotypes, first raw data of day 2–7 in LD were averaged for each single fly. Thereafter, single activity profiles were averaged across all entrained flies of each genotype and smoothed by applying a moving average of 11. For determining the individual free-running period (τ) of rhythmic flies, DD data from day 2–12 were analyzed using χ^2^-periodogram analysis and average period length of each genotype was calculated. To analyze timing of evening activity, raw LD data were converted into 15 minutes bins and evening activity onset was determined after generation of average days for each single fly. Finally, data were averaged across the genotype and tested for statistically significance.

### Immunohistochemistry

To investigate p38 expression and oscillations in nuclear PER in adult *Drosophila* brain, 5–10 days old male flies were entrained to LD 12∶12 for at least 4 days and collected at indicated Zeitgeber Times (ZT; ZT0 indicates lights-on and ZT12 lights-off). To analyze nuclear PER and TIM localization under free-running conditions, flies were first entrained to LD 12∶12 for 4 days followed by 4 days in constant darkness and collected 96 hours after lights-on (ZT1) of the last day LD every 4 hours. Time points of collections were afterwards converted into Circadian Time (CT) according to the onset of activity in free-running flies that were monitored in parallel under the same conditions. Hereby, the activity onset of the flies on day 4 in DD is defined as CT0 and their activity offset as CT12. For light pulse (LP) experiments flies were reared in LD12∶12 for 4 days, subsequently transferred to DD and collected at CT6 and CT18 on day 1 in DD right before as well as after 15 minute light-pulse. Flies were fixed in 4% paraformaldehyde (PFA) in 0.1M phosphate buffer (PB; pH 7.4) with 0.1% Triton X-100 for 2.5 hours. For fixation of flies expressing GFP, no Triton X-100 was used in the PFA solution and fixation time was increased for additional 30 minutes. The fixation step was carried out on a shaker at room temperature and, if necessary, in absence of any light. After fixation flies were rinsed five times for 10 minutes in PB. After dissection 5% normal goat serum (NGS) in PB with 0.5% Triton X-100 was used for blocking samples overnight at 4°C. Next, samples were subsequently incubated with primary antibodies that were diluted in PB with 0.5% Triton X-100, 5% NGS and 0.02% NaN_3_ as follows: chicken anti-GFP 1∶1000 (Abcam), rabbit anti-PER 1∶1000 (kindly provided by R. Stanewsky), rat anti-TIM 1∶1000 (kindly provided by I. Edery), mouse anti-PDF 1∶1000 (Developmental Studies Hybridoma Bank; DSHB), guinea pig anti-VRI 1∶3000 (kindly provided by P. Hardin), goat anti-p38 1∶50 (dN20; Santa Cruz Biotechnology), rabbit anti-p38b 1∶100 (Adachi-Yamada et al., 1999; kindly provided by T. Adachi-Yamada) and rabbit anti-phospho-p38 1∶100 (#4631; Cell Signaling Technology). Goat anti-p38 and rabbit anti-p38b are directed against *Drosophila* p38 and recognize the active (phosphorylated) and inactive (unphosphorylated) forms of p38a and p38b (http://www.scbt.com/datasheet-15714-p38-dn-20-antibody.html; T. Adachi-Yamada personal communication). Rabbit anti-phospho-p38 recognizes human p38 only when dually phosphorylated at Thr180 and/or Tyr182 and does not cross-react with phosphorylated forms of neither p42/44 MAPK nor SAPK/JNK. Due to high-sequence-homology of the p38 phospho sites, the antibody recognizes also *Drosophila* phospho-p38 [32]. Furthermore, its specifity for phospho-p38 has been shown by several studies [69]–[70]. After 24–48 hours primary antibody incubation samples were rinsed five times for 10 minutes in PB with 0.5% Triton X-100 before secondary antibodies were applied. For double or triple immunolabeling Alexa Fluor 488, Alexa Fluor 555 and Alexa Fluor 647 (all from Molecular Probes) were used as secondary antibodies in a dilution of 1∶200 in PB with 5% NGS and 0.5% Triton X-100. After 3 hours at room temperature secondary antibody solution was removed and samples were rinsed five times for 10 minutes in PB with 0.5% Triton X-100. After a final wash step in PB with 0.1% Triton X-100 brains were embedded in Vectashield mounting medium (Vector Laboratories).

### Microscopy and image analysis

The fluorescence signal of immunolabeled brains was visualized using a laser scanning confocal microscope (Zeiss LSM 510 Meta; Carl Zeiss MicroImaging Germany) with a 20× objective. To excite the fluorophores of the secondary antibodies, we used three different diode laser lines 488 nm, 532 nm and 635 nm. In order to avoid bleed through, individual channels were scanned separately, one after another. After confocal stacks of 2 µm thickness were obtained, stacks were subsequently imported into ImageJ to measure staining intensities, to crop the images and to generate overlays. Except of adjustment of brightness and contrast, we performed no other manipulations on the images. To quantify p-p38 staining intensity, both hemispheres of 10 brains per genotype were examined and staining intensity of DN_1a_s was measured using ImageJ as described previously [71]. In order to investigate nuclear translocation of PER in LN_v_s of *UAS-p38b^DN-S^;Pdf-Gal4/+;+* flies and respective controls, we examined 7 brains per ZT and genotype. This time the parameters area, integrated density and mean grey value of defined regions (nucleus, cell body and respective background) were measured and the corrected total cell fluorescence (CTCF) of nucleus as well as cell body was calculated using following formula: CTCF_Nucleus/Cell_ = Integrated density_Nucleus/Cell_ – (Area_Nucleus/Cell_×Mean fluorescence_Background_). Finally, nuclear signal (CTCF_Nucleus_) was normalized to total cell fluorescence (CTCF_Cell_) to determine nuclear translocation of PER in s-LN_v_s and l-LN_v_s.

### RNA extraction and quantitative PCR

To analyze *p38* mRNA expression, 5–10 days old male adult flies were synchronized by LD 12∶12 for 4 days. On the fifth day flies were collected according to ZTs and quickly decapitated on ice. Total RNA from 5 fly heads per genotype and ZT was extracted using the Quick RNA Micro Prep Kit (Zymo Research). cDNA derived from this RNA (using QuantiTect Reverse Transcription Kit from Qiagen) was used as a template for quantitative real-time PCR (qPCR) in combination with the SensiFAST SYBR No-Rox Mix (Bioline) and one of the following primers: 5′-GCCCGTAGACAAATGGAAGGA-3′ and 5′-AACCTGAGCATACGATGGTGG-3′ for p38a, 5′-GAGATGGTCTTCAGCGAGGT-3′ and 5′-AGCATCATTGAACGGAGAGGG-3′ for p38b and 5′-TCTGCGATTCGATGGTGCCCTTAAC-3′ and 5′-GCATCGCACTTGACCATCTGGTTGGC-3′ for *α*-tubulin.

### Western blot analysis

5–10 days old flies were entrained to LD 12∶12 for at least 4 days and collected every 2 hours. To analyze PER cycling, 25 heads of male flies per ZT were homogenized in protein extraction buffer (20 mM HEPES pH 7.5; 100 mM KCl; 5% glycerol; 10 mM EDTA; 0.1% Triton X-100; 20 mM β-glycerophosphate; 0.1 mM Na_3_VO_4_ pH 11) containing a protease inhibitor cocktail (cOmplete Mini EDTA-free; Roche) and loaded onto a 6% gel. SDS-polyacrylamide gel electrophoresis and transfer to nitrocellulose paper were performed according to standard immunoblotting protocols. To minimize differences due to variations in gel electrophoresis and protein blotting, samples of flies with altered p38 levels and respective controls were run and blotted to membrane simultaneously and repeated 4 times. For visualizing daily PER cycling, membranes were incubated in primary and secondary fluorescent antibodies which were diluted in tris-buffered saline with 0.1% Tween-20 (TBST) as follows: rabbit anti-PER 1∶10000 (kindly provided by R. Stanewsky), Alexa Fluor goat-anti-rabbit 680 1∶5000 (Invitrogen). Fluorescent signals were detected using the Odyssey Imaging System (Li-cor Bioscience).

### Protein expression, purification and *in vitro* kinase assays

To express His_6_-p38b, the expression construct was introduced into BL21(DE3)pLYSs competent E. coli cells (Promega) and protein expression was induced at an optical density of ∼0.5 (OD_600_) with 0.3 mM isopropyl-β-D-thiogalactopyranoside (IPTG) for 3 hours at 37°C. After induction cells were pelleted, washed in phosphate-buffered saline (PBS) and pellet was frozen once overnight. Thawed lysate was then solubilized in lysis buffer (50 mM NaH_2_PO_4_; 300 mM NaCl; 10 mM imidazole; 1 mM PMSF; 10 µg/ml leupeptin; pH 8.0) containing protease inhibitor cocktail (complete Mini EDTA-free; Roche) and sonicated 5×5 seconds with short pauses on ice. After sonication Triton X-100 was added to a final concentration of 1% and lysate was centrifuged at 10000 g for 30 minutes at 4°C. Purification of His_6_-p38b protein kinase from supernatant was subsequently carried out by column chromatography using His-Select Nickel Affinity Gel (Sigma) according to manufacturer's protocol. Finally, His_6_-p38b was concentrated and elution buffer (50 mM NaH_2_PO_4_; 300 mM NaCl; 250 mM imidazole) was exchanged by several centrifugation steps (3 minutes at 4000 rpm) using Amicon Ultra centrifugal filter units (MWCO 30 kDa; Sigma) and PBS. For expression of the various GST-PER fusion proteins and GST-p38b, E. coli DH5α containing the appropriate expression plasmids were grown at 37°C to an optical density of 1.2 (OD_600_). Expression was induced by adding IPTG to a final concentration of 0.1 mM accompanied by a reduction of the incubation temperature to 25°C. After 4 hours bacteria were harvested by centrifugation and solubilized in lysis buffer (137 mM NaCl; 2.7 mM KCl; 10 mM Na_2_HPO_4_; 1.8 mM KH_2_PO_4_; 100 mM EDTA; 1% Triton X-100; pH 7.5) supplemented with protease inhibitors (Roche Complete Cocktail and 1 mM PMSF). Resuspended cells were then lysed by sonication and the lysate was cleared by 30 minutes centrifugation at 15000 g and 4°C. After centrifugation, lysates were incubated at 4°C overnight on a rotary wheel with 1.5 ml glutathione sepharose 4B beads (GE Life Sciences) to bind the fusion proteins. The beads were then transferred to a 10 ml Polyprep column (Biorad) and washed once with lysis buffer and thrice with wash buffer (50 mM Tris; 100 mM EDTA; 0.1% Tween-20; pH 7.5). The fusion protein was then eluted from the GSH-Sepharose beads using a 100 mM glutathione solution adjusted to pH 7.5 with 1 M TrisHCl (pH 8.8). Finally the eluate was dialyzed in 5 mM TrisHCl (pH 7.5) for 48 hours and stored at −80°C. To perform non-radioactive phosphorylation assays, 5 µM substrate (GST-PER^1–700^ and GST-PER^658–1218^) was incubated in kinase buffer (50 mM Tris-HCl; 5 mM DTT; 30 mM Mg^2+^; 0.1 mM Na_3_VO_4_) containing 1 mM ATP at 30°C. Kinase assays were initiated by the addition of 1 µM His_6_-p38b and stopped directly after, 30 minutes or 60 minutes after kinase addition by adding equal amount of 2× urea-loading buffer (9 M urea; 20 mM Tris; 190 mM glycine; 1.5 mM EDTA pH 7.5; 1 mM DTT; 0.016% brom phenol blue). After one additional hour incubation at room temperature, protein separation was examined using urea-PAGE and visualized by Coomassie staining, except of some samples that were cut before Coomassie treatment and immunoblotted as described in the western blot section.

Radioactive *in vitro* kinase reactions were conducted in standard kinase buffer (20 mM HEPES pH 7.6, 20 mM MgCl_2_, 10 mM β-glycerophosphate, 0.5 mM Na_3_VO_4_) containing 5 µCi γ-^32^P-ATP per reaction. 20 µg of GST-p38b kinase and of the indicated GST-PER fusion protein or GST as a control were added to each reaction and incubated at 30°C for 30 minutes. The proteins were then separated by SDS-PAGE and phosphorylation was detected by autoradiography. Gels were stained with Coomassie brilliant blue to visualize total protein amounts in the various samples. Relative levels of phosphorylation were quantified using the open source software Fiji [72].

### Statistics

All data were tested for normal distribution by a one-sample Kolmogorov–Smirnov test (Lilliefors). Normally distributed data were statistically compared by a one-way analysis of variance (ANOVA) followed by a post-hoc test with Bonferroni correction for pairwise comparison. Not normally distributed data were compared by a Kruskal–Wallis test followed by Wilcoxon analysis (Systat 11, SPSS, Chicago, IL; USA) with Bonferroni correction. Data were regarded as significantly different at p<0.05 and as highly significant at p<0.001.

## Supporting Information

Figure S1 Expression pattern of p38 MAPK in adult male *Drosophila melanogaster* brains investigated by *p38b-Gal4*-driven GFP (A–C) and a *Drosophila* antibody recognizing all forms of *Drosophila* p38 (D–G). A–C: GFP displayed a broad expression pattern, showing strong expression, amongst others, in the pars intercerebralis (PI), mushroom body (MB), suboesophageal ganglion (SOG) as well as in the cortical area between the inner margin of the medulla (ME) and the central brain (CB). Co-labeling of GFP (green)-expressing cells with anti-PER (magenta) and anti-PDF (blue) revealed p38b expression in 4 l-LN_v_s (white stars in C2). B1-3 represent an image stack showing s-LN_v_s and LN_d_s, C1-3 display close-ups of the l-LN_v_s. D–G: Anterior (D and E) and posterior (F and G) view of *Canton S* wildtype brains labeled with the *Drosophila* anti-p38b antibody (green) and two clock specific antibodies - anti-VRI (magenta) and anti-PDF (blue) - showing a similar widespread staining pattern as did *p38b-Gal4;UAS-GFP^S65T^* flies (D and E compared to A). Furthermore, p38b staining was most prominent in regions of lateral neurons (white arrowheads in D and E1-3; for a more magnified illustration of LN_v_s see Fig. 1A–C) as well as in the entire cortex of the dorsal brain (white arrowheads in F) including the region of the dorsal neurons (G1-3). Scale bar = 10 µm.
(TIF)

Figure S2 Expression of active p38 in DN_1a_s at ZT21 in *Canton S* wildtype, *p38b^Δ45^* and *p38a^Δ1^* flies. Both *p38* null mutants displayed a significant reduction of p-p38 to 50% of wildtype level (p<0.05). Colored bars represent p-p38 levels of the genotypes normalized to the wildtype level. Error bars show SEM. Significant differences (p<0.05) are indicated by *.
(TIF)

Figure S3 Number of p-p38 positive DN_1a_s per wildtype brain hemisphere in course of a day. Daily variations in p38 activity in DN_1a_s is not solely attributed to decreased or increased total p-p38 levels, it's additionally the oscillating number of p-p38 stained DN_1a_s per hemisphere that contributes. Even if in some cases not all DN_1a_s of a brain hemisphere showed p-p38 staining during the night (ZT13-21), the average number of p-p38 positive DN_1a_s was significantly higher than during the day. Colored bars represent average p-p38 positive DN_1a_ per hemisphere. Error bars show SEM. Significant differences (p<0.05) are indicated by *.
(TIF)

Figure S4 *p38a* and *p38b* mRNA expression in *w;+;UAS-p38a^RNAi^/da-Gal4* (A) and *w;UAS-p38b^RNAi^/+;da-Gal4/+* (B) compared to respective controls. Expression data of three biological replicates were averaged within the genotype and normalized to wildtype level. A: Quantitative real-time PCR revealed a high significant reduction in *p38a* mRNA in *w;+;UAS-p38a^RNAi^/da-Gal4*, confirming the effectiveness of the *p38a^RNAi^* transgene (p<0.001). B: Furthermore, significant reduction of *p38b* mRNA to 50% of wildtype level in *w;UAS-p38b^RNAi^/+;da-Gal4/+* additionally proved the effectiveness of the *p38b^RNAi^* transgene (p<0.05). Error bars show SEM. Significant differences (p<0.05) are indicated by *, highly significant differences (p<0.001) by **.
(TIF)

Figure S5 Locomotor activity rhythms of flies expressing a *UAS-p38b kinase-dead* transgene (*UAS-p38b^KD^*) in *Drosophila* clock neurons and respective controls. In LD, both experimental lines, *w;UAS-p38b^KD^/tim(UAS)-Gal4*;*UAS-p38b^DN-S^/+* (upper panels in A) and *w;UAS-p38b^KD^/Pdf-Gal4*;*UAS-p38b^DN-S^/+* (upper panels in B), showed a diurnal activity pattern with activity bouts around lights-on and lights-off, but a significant later evening activity onset than control flies. This tendency proceeded in a significantly prolonged free-running rhythm when flies were transferred to DD (lower panels in A and B). For recording and processing of activity data as well as for figure labeling see Figure 3.
(TIF)

Figure S6 PER and TIM clock protein cycling in p38b knockdown flies in DD. Nuclear PER (red) and TIM (blue) staining intensity was evaluated on the 4^th^ day in DD in the s-LN_v_s after down-regulation of p38b with *Pdf-gal4* (p38b RNAi = *dicer2;UAS-p38b^RNAi^/Pdf-Gal4;+* flies). *UAS-p38b^RNAi^;+* flies served as control. Interestingly, immunostainings revealed that the molecular cycling still persists in *dicer2;UAS-p38b^RNAi^/Pdf-Gal4;+* flies. However, the phase of the clock protein oscillation was delayed, which is in line with the long free-running period of these flies. Grey bars on top of the graphs indicate the subjective day of the flies, that starts with their activity (act.) onset ( = Circadian Time (CT) 0). Black bars indicate the subjective night of the flies that begins with the activity offset ( = CT 12). For better clarity 12 hours before and after the measured day are repeated to the left and the right (dotted curves). Red and blue arrows point to peaks in nuclear PER and TIM, respectively.
(TIF)

Figure S7 Locomotor activity rhythms of flies overexpressing wildtype p38b (*p38b^+^*) and respective controls. Flies overexpressing p38b either in TIM-positive (*dicer2;tim(UAS)-Gal4/+;UAS-p38a^RNAi^/+*, A) or PDF-positive clock neurons (*dicer2;Pdf-Gal4/+;UAS-p38a^RNAi^/+*, B) showed wildtype-like locomotor behavior in LD with activity bouts around lights-on and lights-off. However, evening activity onset of both lines was significantly delayed compared to controls (upper panels in A and B) and resulted in a prolonged free-running period after transfer to constant darkness. For recording and processing of activity data as well as for figure labeling see Figure 3.
(TIF)

Figure S8 *p38a* (A) and *p38b* (B) mRNA expression in *Canton S* wildtype, *p38b^Δ45^* and *p38a^Δ1^* heads. Expression data of three biological replicates per genotype were averaged within the genotype and normalized to wildtype level. Quantitative real-time PCR clearly confirmed our *p38a* null (A) and *p38b* null (B) phenotypes (p<0.05 and p<0.001 respectively). In addition there was no compensatory effect on the transcription of one p38 isoform, when the other was missing. Error bars show SEM. Significant differences (p<0.05) are indicated by *, highly significant differences (p<0.001) by **.
(TIF)

Figure S9 *Drosophila* PER contains two p38 consensus phosphorylation sites. Online research (http://www.kinexus.ca/pdf/graphs_charts/ProteinSerKinaseSpecificity.pdf) and amino acid sequence comparison revealed that *Drosophila* PER contains two predicted p38 consensus phosphorylation sites (PXS*P): Ser661 and Ser975. The latter has not been described as phosphorylation site so far. In contrast, there is evidence that a proline-directed kinases, a family also p38 belongs to, phosphorylates PER at Ser661 and thereby primes it for further phosphorylation at Ser657 by SGG. Black characters represent *Drosophila* PER amino acid sequence, red characters represent predicted p38 MAPK consensus phosphorylation sites and stars indicate previous identified PER phosphorylation sites.
(TIF)
