# Supplementary figures and images for: The MAP Kinase p38 Is Part of Drosophila melanogaster's Circadian Clock
Source: PLoS Genet. 2014 Aug 21;10(8):e1004565. doi: 10.1371/journal.pgen.1004565 (PMC4140665; doi:10.1371/journal.pgen.1004565)

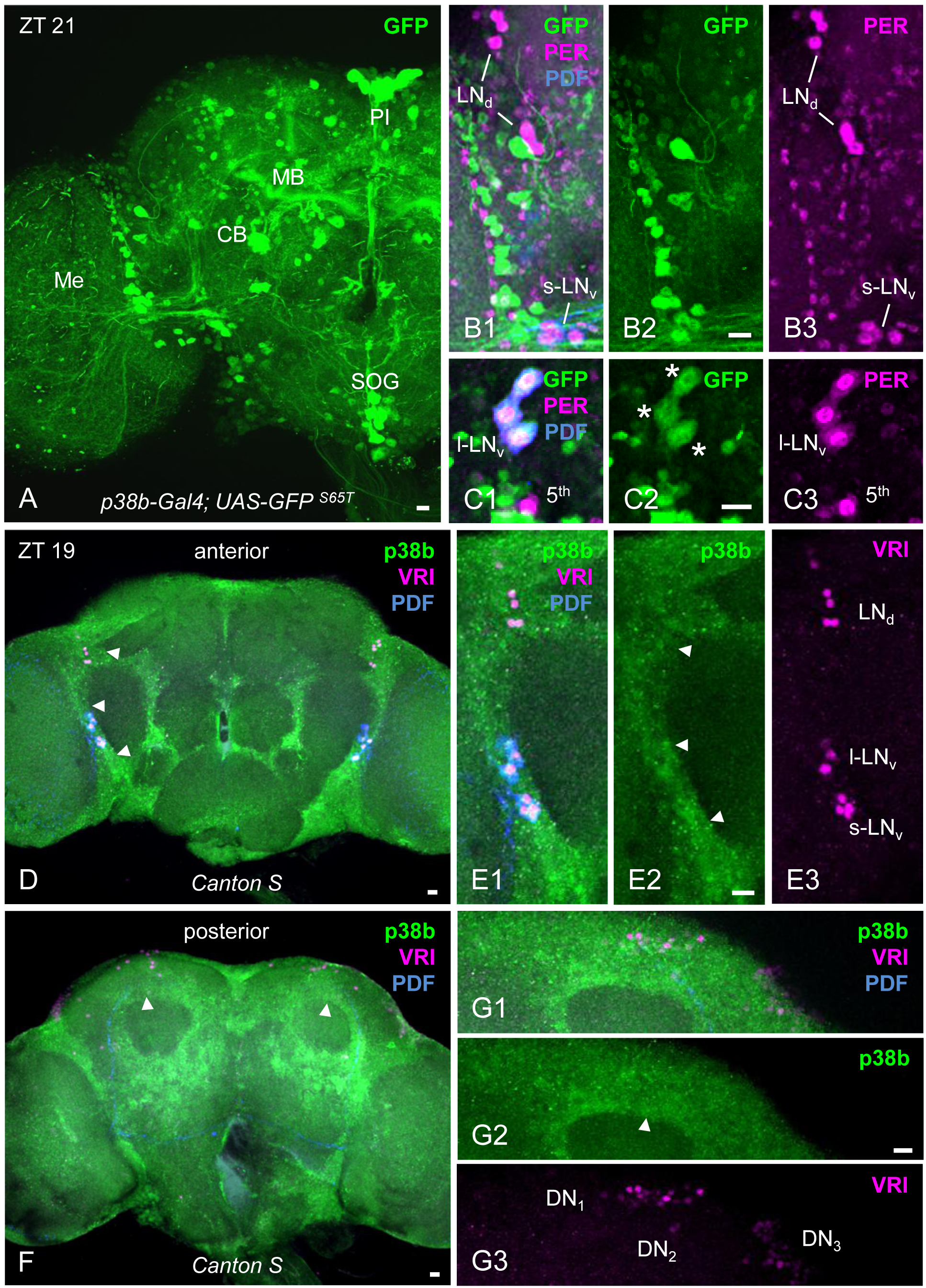

Supplement: Figure S1 — Expression pattern of p38 MAPK in adult male Drosophila melanogaster brains investigated by p38b-Gal4-driven GFP (A–C) and a Drosophila antibody recognizing all forms of Drosophila p38 (D–G). A–C: GFP displayed a broad expression pattern, showing strong expression, amongst others, in the pars intercerebralis (PI), mushroom body (MB), suboesophageal ganglion (SOG) as well as in the cortical area between the inner margin of the medulla (ME) and the central brain (CB). Co-labeling of GFP (green)-expressing cells with anti-PER (magenta) and anti-PDF (blue) revealed p38b expression in 4 l-LNvs (white stars in C2). B1-3 represent an image stack showing s-LNvs and LNds, C1-3 display close-ups of the l-LNvs. D–G: Anterior (D and E) and posterior (F and G) view of Canton S wildtype brains labeled with the Drosophila anti-p38b antibody (green) and two clock specific antibodies - anti-VRI (magenta) and anti-PDF (blue) - showing a similar widespread staining pattern as did p38b-Gal4;UAS-GFPS65T flies (D and E compared to A). Furthermore, p38b staining was most prominent in regions of lateral neurons (white arrowheads in D and E1-3; for a more magnified illustration of LNvs see Fig. 1A–C) as well as in the entire cortex of the dorsal brain (white arrowheads in F) including the region of the dorsal neurons (G1-3). Scale bar = 10 µm. (TIF) [file pgen.1004565.s001.tif]

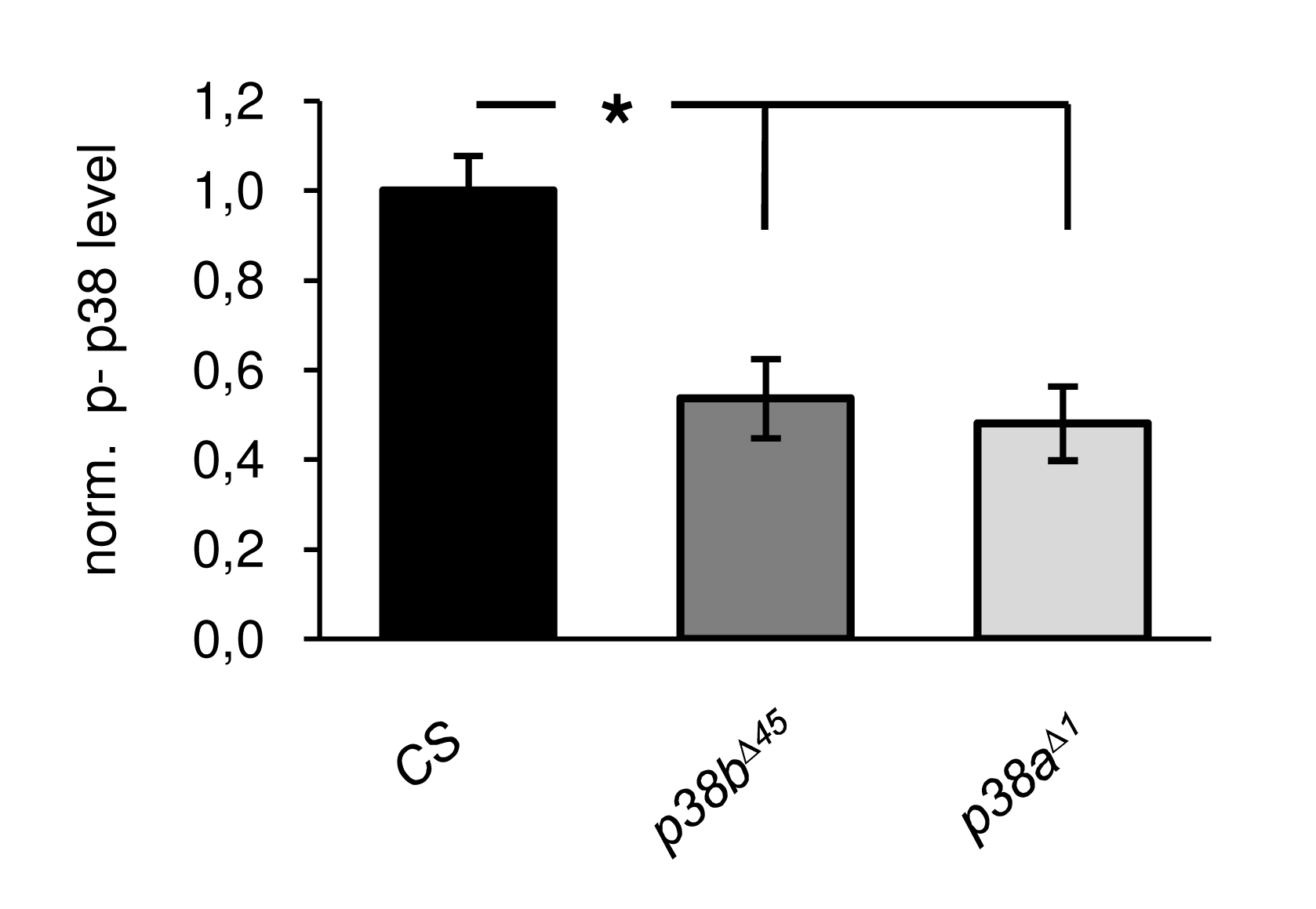

Supplement: Figure S2 — Expression of active p38 in DN1as at ZT21 in Canton S wildtype, p38bΔ45 and p38aΔ1 flies. Both p38 null mutants displayed a significant reduction of p-p38 to 50% of wildtype level (p<0.05). Colored bars represent p-p38 levels of the genotypes normalized to the wildtype level. Error bars show SEM. Significant differences (p<0.05) are indicated by *. (TIF) [file pgen.1004565.s002.tif]

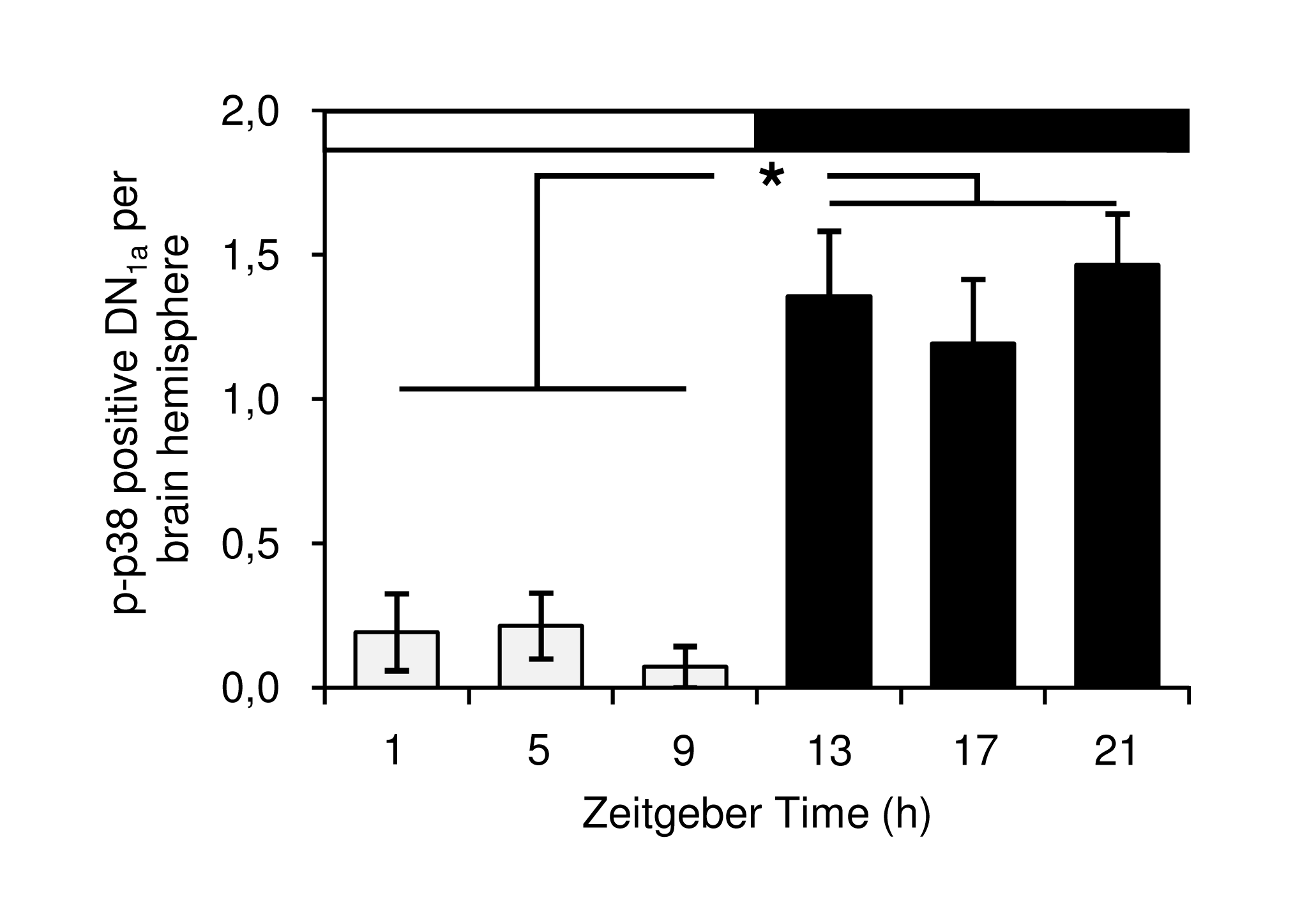

Supplement: Figure S3 — Number of p-p38 positive DN1as per wildtype brain hemisphere in course of a day. Daily variations in p38 activity in DN1as is not solely attributed to decreased or increased total p-p38 levels, it's additionally the oscillating number of p-p38 stained DN1as per hemisphere that contributes. Even if in some cases not all DN1as of a brain hemisphere showed p-p38 staining during the night (ZT13-21), the average number of p-p38 positive DN1as was significantly higher than during the day. Colored bars represent average p-p38 positive DN1a per hemisphere. Error bars show SEM. Significant differences (p<0.05) are indicated by *. (TIF) [file pgen.1004565.s003.tif]

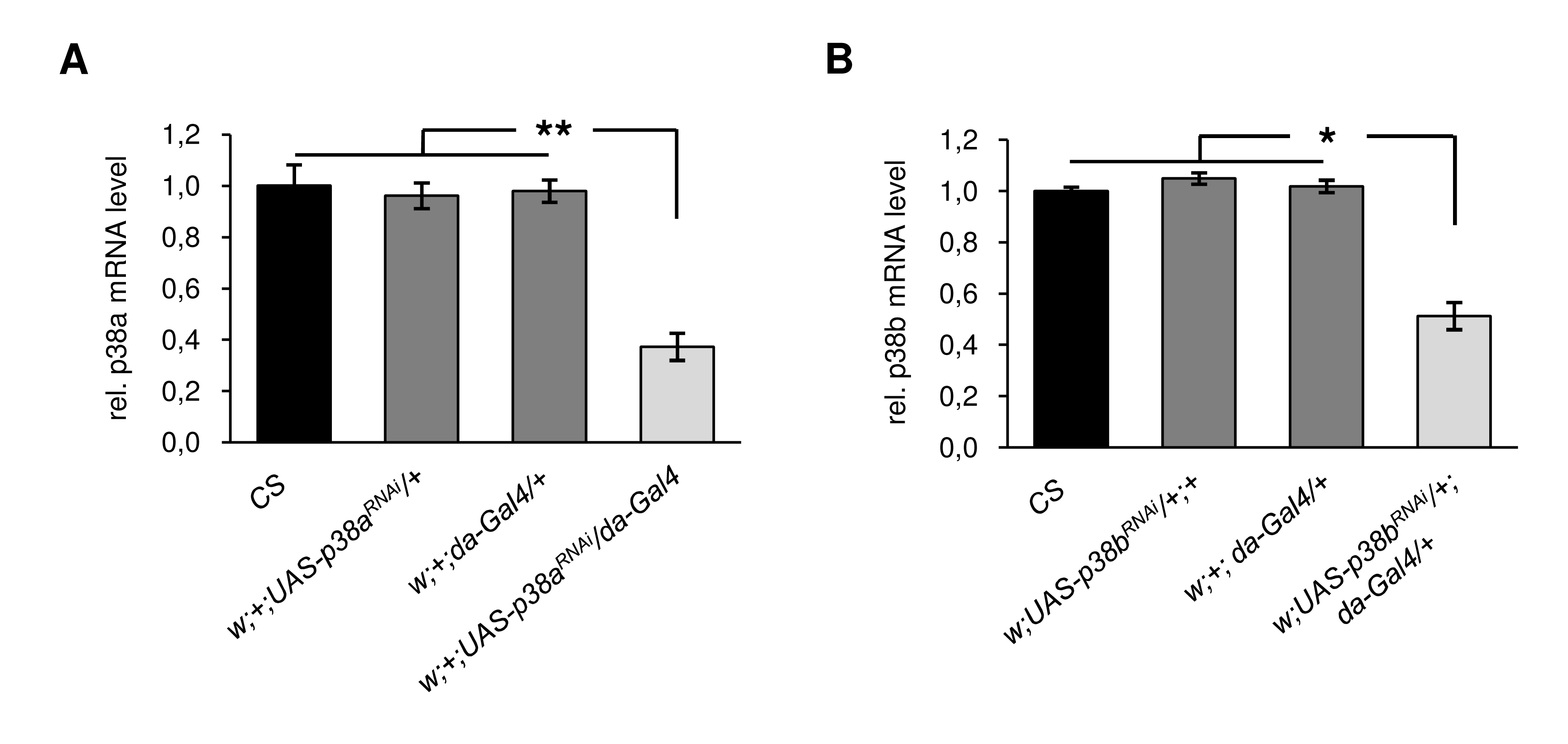

Supplement: Figure S4 — p38a and p38b mRNA expression in w;+;UAS-p38aRNAi/da-Gal4 (A) and w;UAS-p38bRNAi/+;da-Gal4/+ (B) compared to respective controls. Expression data of three biological replicates were averaged within the genotype and normalized to wildtype level. A: Quantitative real-time PCR revealed a high significant reduction in p38a mRNA in w;+;UAS-p38aRNAi/da-Gal4, confirming the effectiveness of the p38aRNAi transgene (p<0.001). B: Furthermore, significant reduction of p38b mRNA to 50% of wildtype level in w;UAS-p38bRNAi/+;da-Gal4/+ additionally proved the effectiveness of the p38bRNAi transgene (p<0.05). Error bars show SEM. Significant differences (p<0.05) are indicated by *, highly significant differences (p<0.001) by **. (TIF) [file pgen.1004565.s004.tif]

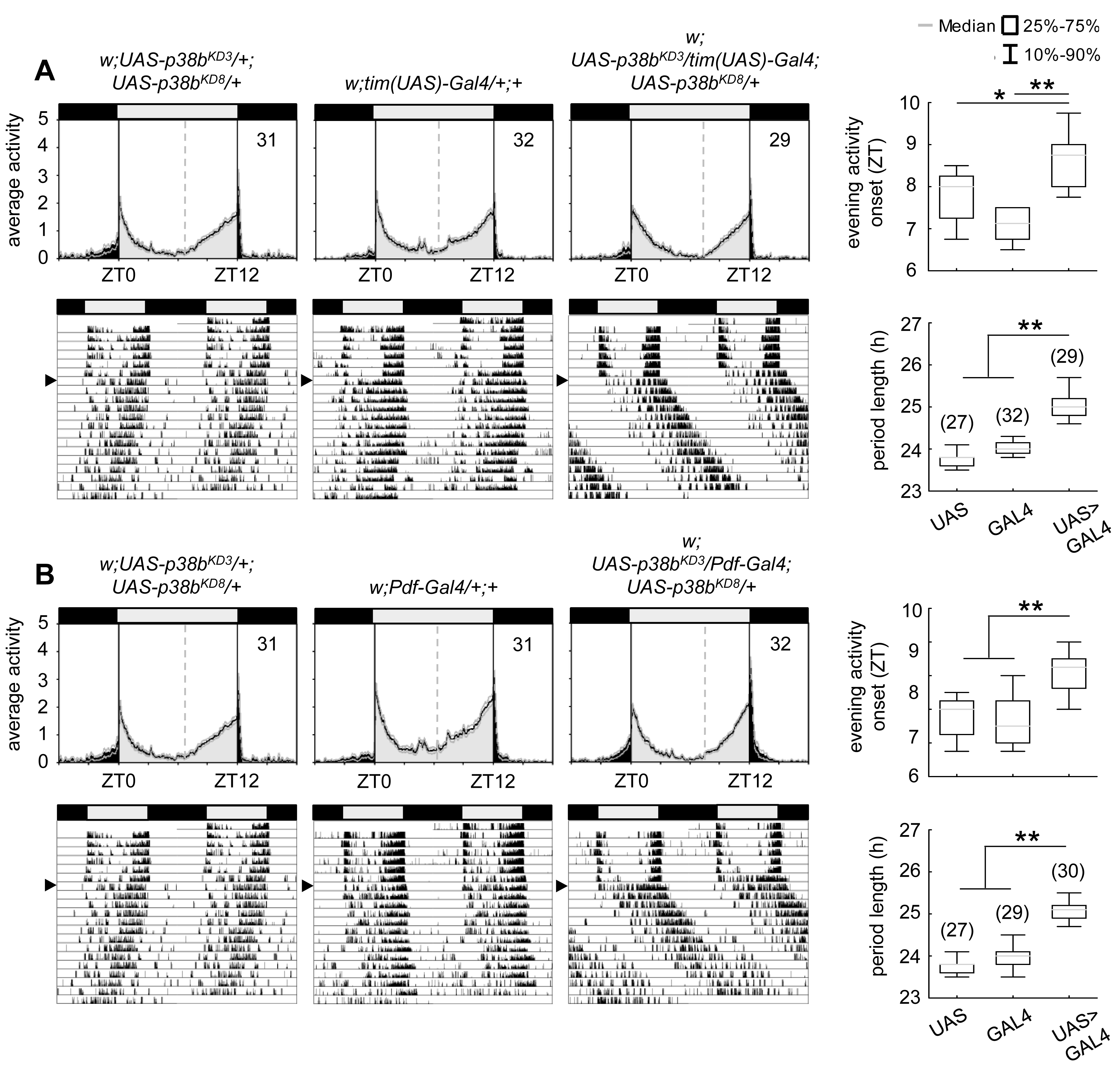

Supplement: Figure S5 — Locomotor activity rhythms of flies expressing a UAS-p38b kinase-dead transgene (UAS-p38bKD) in Drosophila clock neurons and respective controls. In LD, both experimental lines, w;UAS-p38bKD/tim(UAS)-Gal4;UAS-p38bDN-S/+ (upper panels in A) and w;UAS-p38bKD/Pdf-Gal4;UAS-p38bDN-S/+ (upper panels in B), showed a diurnal activity pattern with activity bouts around lights-on and lights-off, but a significant later evening activity onset than control flies. This tendency proceeded in a significantly prolonged free-running rhythm when flies were transferred to DD (lower panels in A and B). For recording and processing of activity data as well as for figure labeling see Figure 3. (TIF) [file pgen.1004565.s005.tif]

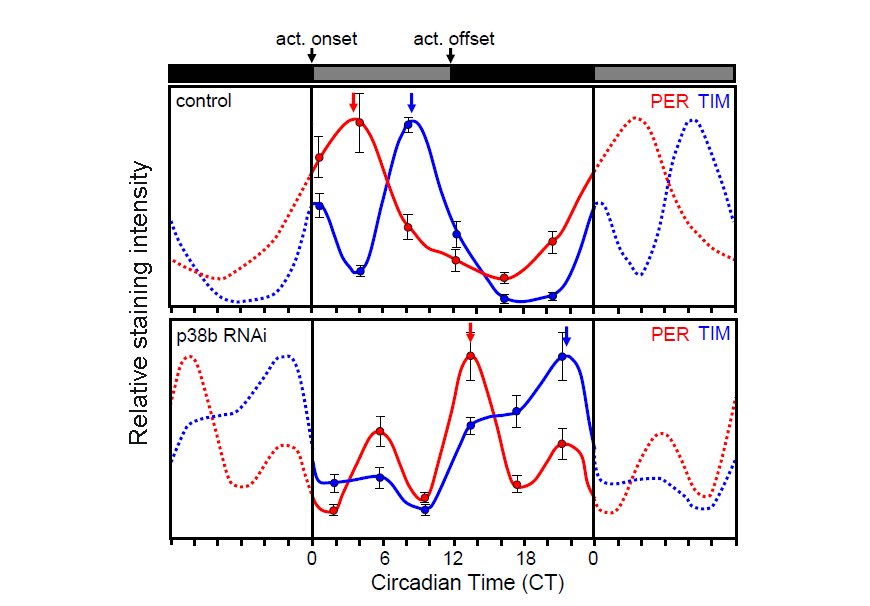

Supplement: Figure S6 — PER and TIM clock protein cycling in p38b knockdown flies in DD. Nuclear PER (red) and TIM (blue) staining intensity was evaluated on the 4th day in DD in the s-LNvs after down-regulation of p38b with Pdf-gal4 (p38b RNAi = dicer2;UAS-p38bRNAi/Pdf-Gal4;+ flies). UAS-p38bRNAi;+ flies served as control. Interestingly, immunostainings revealed that the molecular cycling still persists in dicer2;UAS-p38bRNAi/Pdf-Gal4;+ flies. However, the phase of the clock protein oscillation was delayed, which is in line with the long free-running period of these flies. Grey bars on top of the graphs indicate the subjective day of the flies, that starts with their activity (act.) onset ( = Circadian Time (CT) 0). Black bars indicate the subjective night of the flies that begins with the activity offset ( = CT 12). For better clarity 12 hours before and after the measured day are repeated to the left and the right (dotted curves). Red and blue arrows point to peaks in nuclear PER and TIM, respectively. (TIF) [file pgen.1004565.s006.tif]

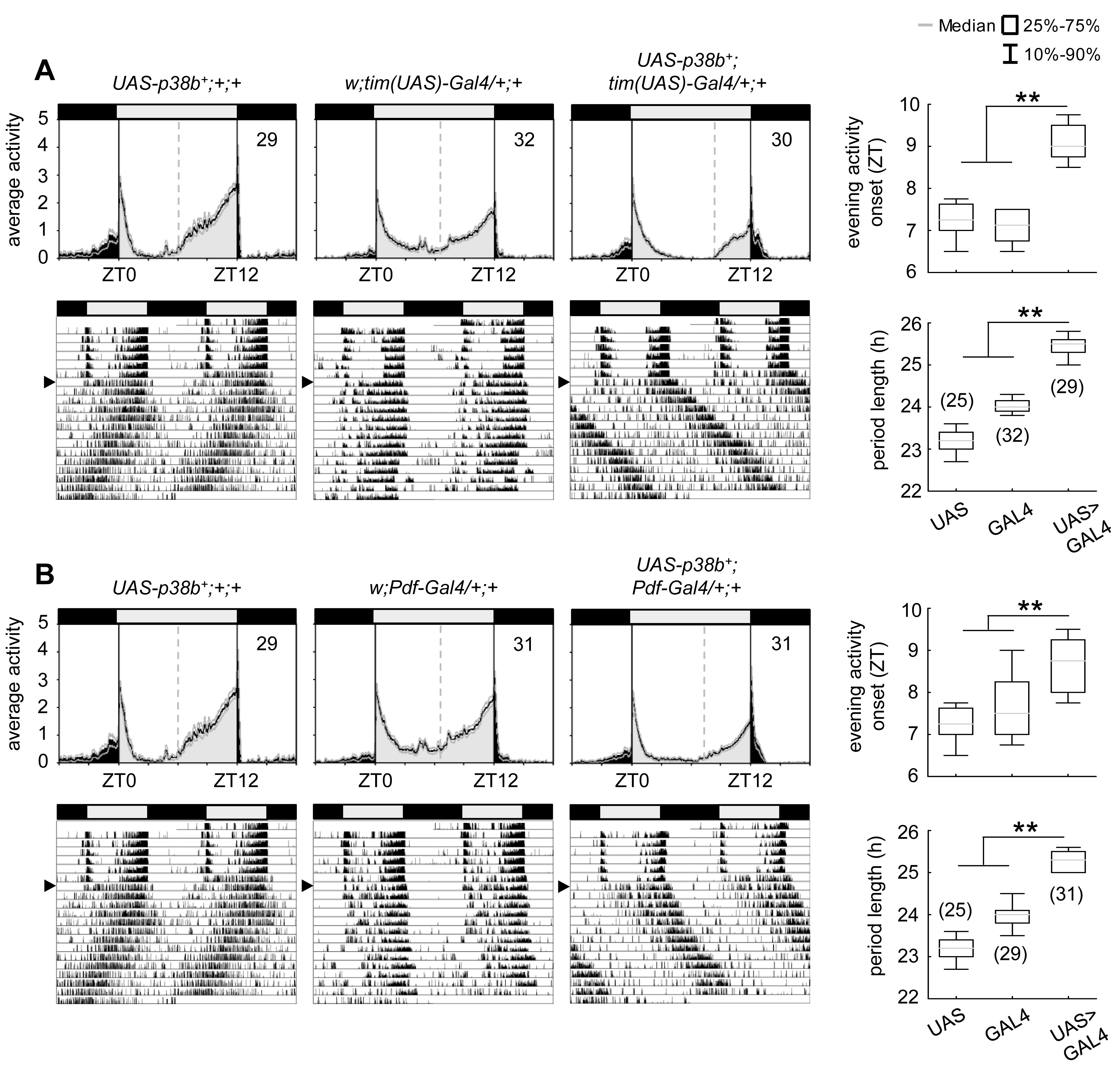

Supplement: Figure S7 — Locomotor activity rhythms of flies overexpressing wildtype p38b (p38b+) and respective controls. Flies overexpressing p38b either in TIM-positive (dicer2;tim(UAS)-Gal4/+;UAS-p38aRNAi/+, A) or PDF-positive clock neurons (dicer2;Pdf-Gal4/+;UAS-p38aRNAi/+, B) showed wildtype-like locomotor behavior in LD with activity bouts around lights-on and lights-off. However, evening activity onset of both lines was significantly delayed compared to controls (upper panels in A and B) and resulted in a prolonged free-running period after transfer to constant darkness. For recording and processing of activity data as well as for figure labeling see Figure 3. (TIF) [file pgen.1004565.s007.tif]

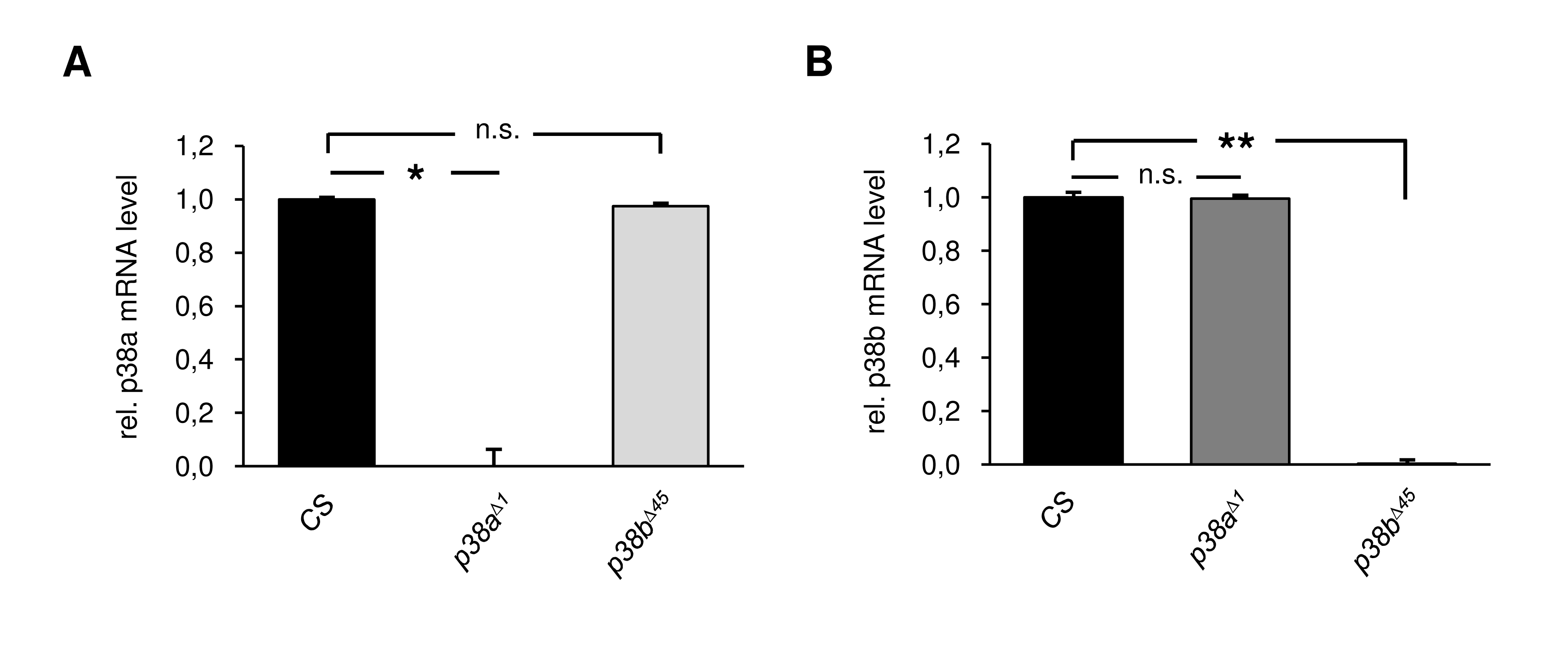

Supplement: Figure S8 — p38a (A) and p38b (B) mRNA expression in Canton S wildtype, p38bΔ45 and p38aΔ1 heads. Expression data of three biological replicates per genotype were averaged within the genotype and normalized to wildtype level. Quantitative real-time PCR clearly confirmed our p38a null (A) and p38b null (B) phenotypes (p<0.05 and p<0.001 respectively). In addition there was no compensatory effect on the transcription of one p38 isoform, when the other was missing. Error bars show SEM. Significant differences (p<0.05) are indicated by *, highly significant differences (p<0.001) by **. (TIF) [file pgen.1004565.s008.tif]

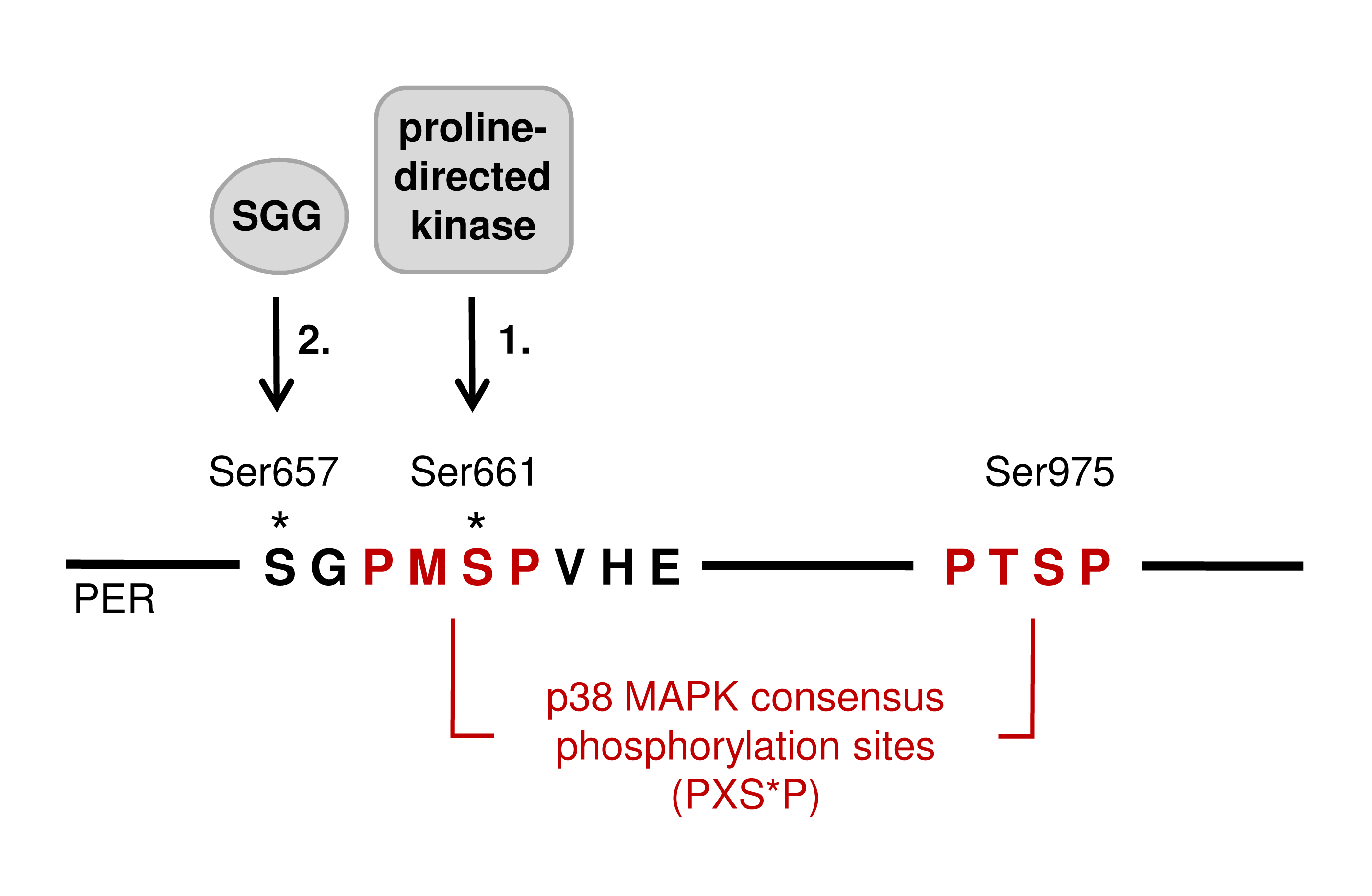

Supplement: Figure S9 — Drosophila PER contains two p38 consensus phosphorylation sites. Online research (http://www.kinexus.ca/pdf/graphs_charts/ProteinSerKinaseSpecificity.pdf) and amino acid sequence comparison revealed that Drosophila PER contains two predicted p38 consensus phosphorylation sites (PXS*P): Ser661 and Ser975. The latter has not been described as phosphorylation site so far. In contrast, there is evidence that a proline-directed kinases, a family also p38 belongs to, phosphorylates PER at Ser661 and thereby primes it for further phosphorylation at Ser657 by SGG. Black characters represent Drosophila PER amino acid sequence, red characters represent predicted p38 MAPK consensus phosphorylation sites and stars indicate previous identified PER phosphorylation sites. (TIF) [file pgen.1004565.s009.tif]
